# Multispectral infrared-to-full-color upconversion expanding human vision

**DOI:** 10.1126/sciadv.aed0245

**Published:** 2026-07-29

**Authors:** Chengchang Fu, Jintao Zou, Xiaoxue Yang, Qun Hao, Xin Tang, Ge Mu

**Affiliations:** ^1^ School of Optics and Photonics, Beijing Institute of Technology, Beijing 100081, China.; ^2^ College of Science, Minzu University of China, Beijing, 100081, China.; ^3^ Beijing Key Laboratory for Precision Optoelectronic Measurement Instrument and Technology, Beijing, 100081, China.; ^4^ Yangtze Delta Region Academy of Beijing Institute of Technology, Jiaxing, 314019, China.

## Abstract

The human visual system is inherently blind to infrared radiation due to the insufficient energy of infrared photons to trigger the photoisomerization of the retinal chromophore, resulting in the loss of over half of the solar spectrum. Here, we report a colloidal quantum dot (CQD)-based infrared-to-visible upconverter that enables ultrasensitive upconversion of multispectral infrared light radiation as full-color visible vision. The photon-energy-selective excitonic transitions in quantum-confined states and the photon flux, respectively, enable infrared wavelength- and intensity-dependent number of photogenerated carriers. A dual-emissive-layer organic architecture with the strategically engineered hole-injection barrier could route these carriers into distinct color emission channels, resulting in correlated wavelength/intensity-to-color/luminance mapping. This paradigm shift yields a discrimination sensitivity for subtle infrared variations exceeding more than two orders of magnitude higher than conventional single-color modes, capitalizing on the human eye’s intrinsic superiority in chromatic differentiation over only brightness contrast. The resulting upconverter exhibits broadband detection extending beyond 2 μm, luminance exceeding 700 cd m^−2^, and a photon-to-photon conversion efficiency of 3.85%. The upconverter could be applied as a lightweight, semi-transparent wearable eyeglass that projects multispectral infrared as full-color vision directly onto the retina. Besides, upconverters bound to light-sensitive proteins, potentially as an implantable retinal photoreceptor, confer innate infrared vision. By surpassing the evolutionary constraints of natural vision, this work establishes a versatile foundation for next-generation visual prosthetics and human-integrated sensory expansion.

## Teaser

Multispectral infrared-to-full-color visible upconversion expands human vision beyond natural limits.

## INTRODUCTION

The human eye, confined by the thermodynamic limit of opsin photopigments, is blind to over half of the Sun’s radiant energy residing in the infrared spectrum (>700 nm) because infrared photons lack sufficient energy (<∼1.6 eV) to reliably trigger the conformational changes required for phototransduction (*1*, *2*). Yet harnessing this imperceptible radiation is critical, as infrared perception unlocks a vast reservoir of inaccessible information, enabling critical functions like navigation in darkness and deep-biological tissue visualization, where our natural vision is utterly inadequate (*3*–*6*).

Recent advances, including ocular injectable photoreceptor-binding upconversion nanoparticles converting infrared light into visible emissions (*7*, *8*), wearable lenses with nonlinear upconversion projecting infrared-transformed visible light onto cornea (*9*, *10*), and photovoltaic nanowire networks achieving broad spectral sensitivity as a retinal nanoprosthesis (*11*), have demonstrated infrared vision capabilities. However, they remain fundamentally constrained to narrow near-infrared (NIR) spectral response and rely on high-intensity laser excitation incompatible with natural infrared radiation, and mostly monochromatic output lacking infrared spectral discrimination (*12*).

Integrated photonic upconverter transcends the fundamental limitations by leveraging a linear photon-to-electron-to-photon conversion process through coupling an infrared photodetector (PD) and a visible light-emitting diode (LED) (*13*–*17*). The spectral freedom arises from selecting infrared photodetecting materials with tailored bandgaps, such as inorganic materials (*18*, *19*) for NIR, organic materials (*13*, *14*, *20*–*25*) for NIR and shortwave infrared (SWIR), and colloidal quantum dots (CQDs) (*16*, *26*–*30*) for extended SWIR and midwave infrared (MWIR), bypassing the fixed energy levels of upconversion nanoparticles. Besides, unlike the inherently low probability of multi-photon interactions in nonlinear upconversion and forcing reliance on high-power infrared lasers, linear upconverters achieve nearly 30% efficiency by sequential high-gain photodetection and efficient electroluminescent conversion (*17*, *22*, *30*). Furthermore, the decoupled photon-to-electron-to-photon cascade allows precise bandgap engineering of infrared photodetectors, coupling with tunable energy alignment of multichromatic emitters to translate distinct infrared wavelengths into discriminable visible colors (*31*). Nevertheless, so far, photonic linear upconverters have never been developed for multispectral infrared full-color visualization and integration with the human eye to enable infrared vision.

Herein, we present a mercury telluride (HgTe) CQD-based infrared-to-visible upconverter capable of upconversion of multispectral infrared radiation spanning the NIR to SWIR regions into full-color vision. The device architecture leverages the quantized energy level states of HgTe CQDs in synergy with a dual-emissive-layer organic light-emitting diode (OLED), engineered via precise band alignment and strategically engineered hole-trapping barriers. This design enables the emitted visible color and luminance to depend jointly on the incident infrared wavelength and intensity. This upconversion yields a discrimination sensitivity to subtle infrared variations exceeding two orders of magnitude higher than conventional single-color modes, capitalizing on the human eye’s intrinsic superiority in chromatic differentiation over simple luminance contrast. The upconverter further delivers broadband infrared response beyond 2 μm, a luminance exceeding 700 cd m^−2^, and a photon conversion efficiency of 3.85%.

**Fig. 1.**
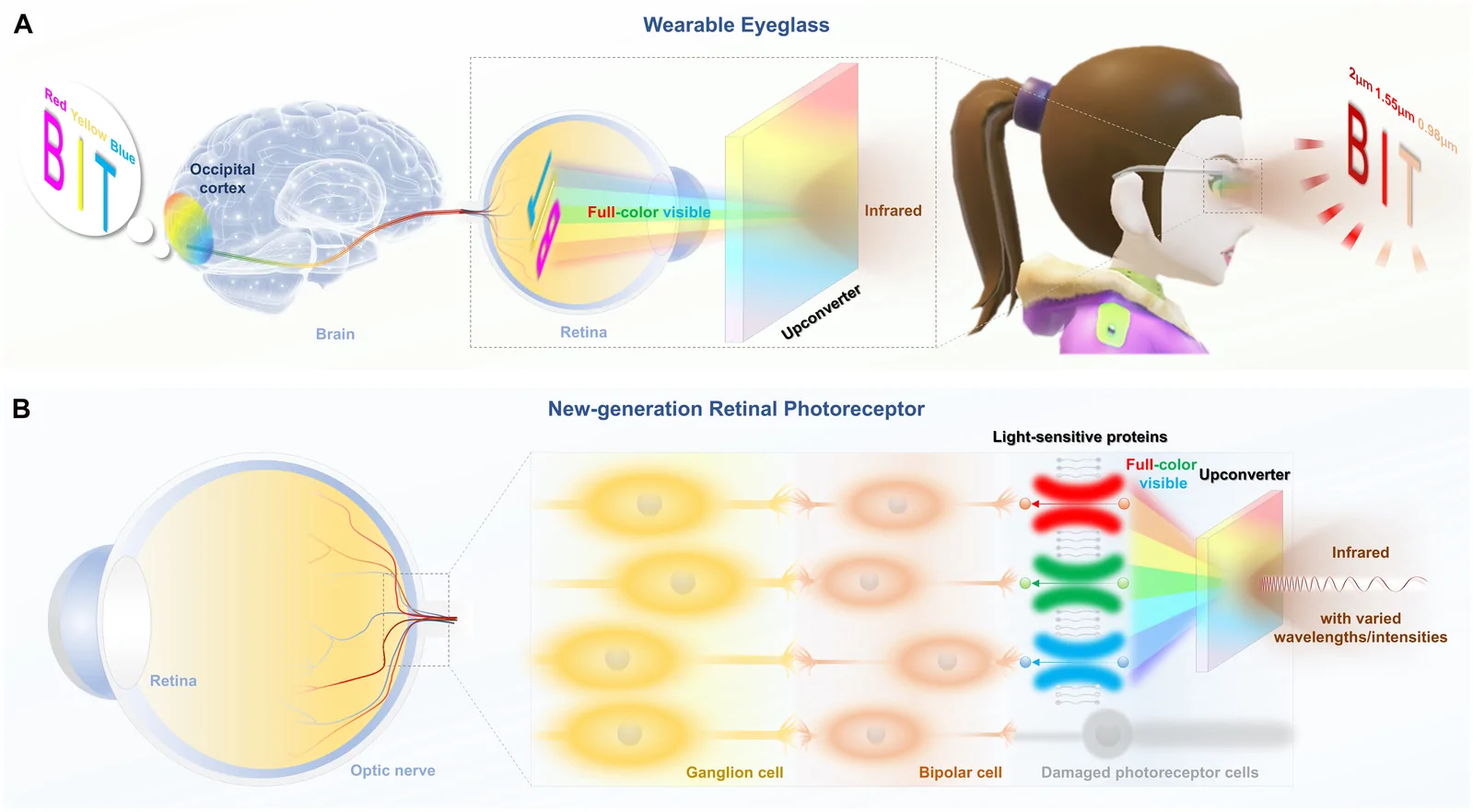
Conceptual diagram. The infrared-to-full-color upconverter is applied as (**A**) the wearable eyeglass and (**B**) the new-generation retinal photoreceptor.

Notably, the upconverter supports dual-mode integration into biological visual systems. As a lightweight (23 g), semi-transparent wearable eyeglass (active area: 3.57 cm^2^), it achieves high-resolution infrared imaging that projects infrared spectral information as full-color visible patterns onto the retina, enabling intuitive infrared vision without obstructing natural sight (Fig. 1A). Interestingly, upconverters bound to light-sensitive proteins could serve as an implantable new-generation retinal photoreceptor, where the upconverter transforms infrared light into visible light emissions that stimulate light-sensitive proteins on retinal neurons to bypass damaged photoreceptor cells and potentially restore visual function across both the visible and infrared spectra (Fig. 1B) (*32*, *33*). Electrophysiological validation in mice and humans confirms successful infrared perception mediated by the device. By surpassing the evolutionary boundaries of biological photoreception, this technology paves the way for next-generation visual prosthetics, high-fidelity augmented reality, molecularly sensitive substance identification, and robust navigation in degraded visual environments.

## RESULTS

### Design rationale and operational mechanism

**Fig. 2.**
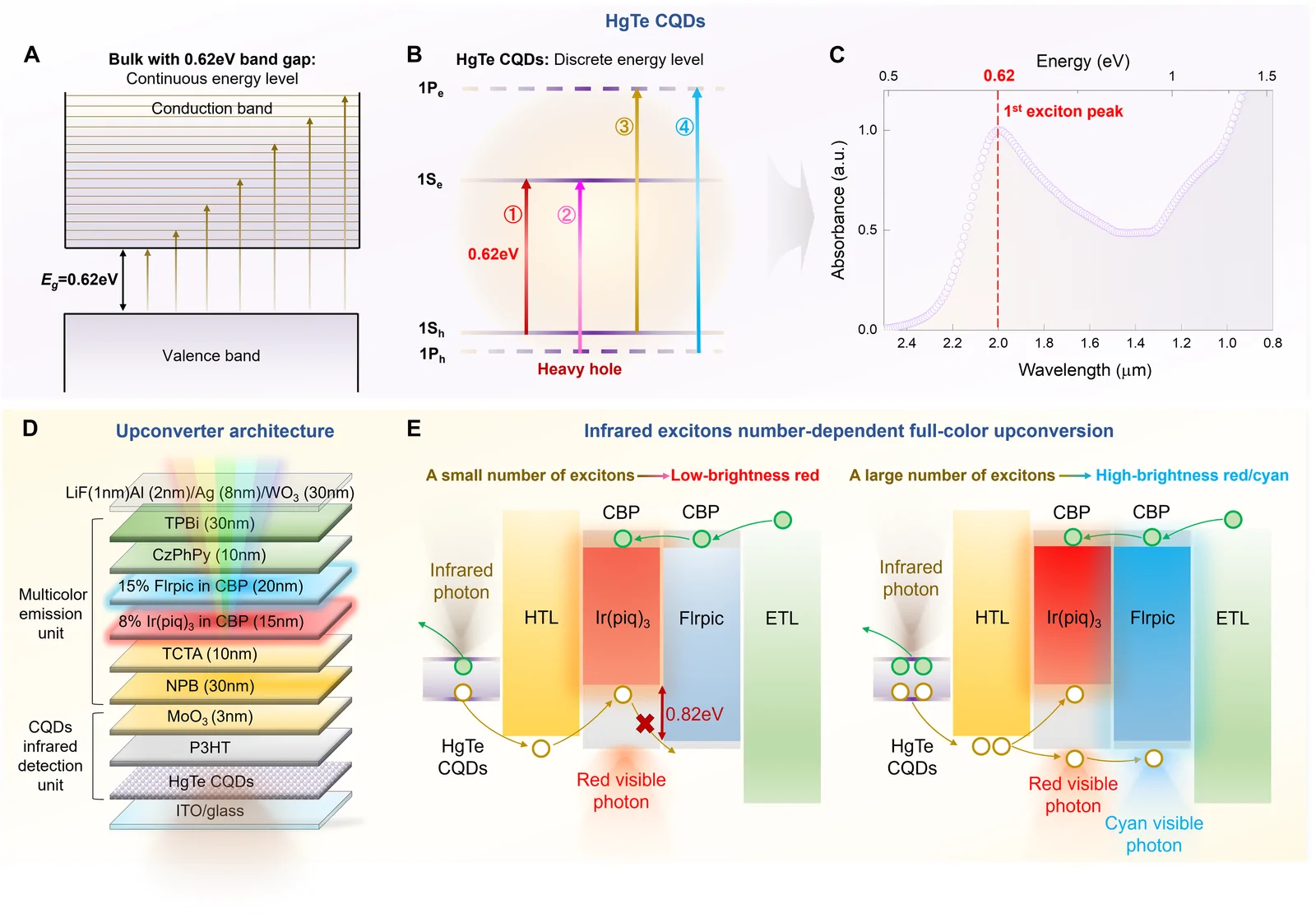
Design rationale and operational mechanism. Energy level diagram of (**A**) bulk material and (**B**) HgTe CQDs. (**C**) Absorption spectra of the HgTe CQDs. (**D**) Structure diagram of the integrated upconverter. (**E**) Energy band diagram of infrared excitons number-dependent full-color upconversion.

Unlike bulk with continuous bands (Fig. 2A) or HgTe CQDs with a wide range of size distributions lacking distinct excitonic features, monodisperse HgTe CQDs (3.94 ± 0.21 nm diameter, fig. S1) feature discrete heavy-hole-dominated energy levels enabling photon-energy-selective excitonic transitions. The valence band is dominated by heavy-hole states with a large effective mass, whereas the conduction band comprises light effective mass states (*34*, *35*). The corresponding energy level diagram based on k·p calculations and spectroscopic studies supported by the references (*34*, *35*) is presented in Fig. 2B. As shown in Fig. 2C, the absorption spectrum of the HgTe CQDs exhibits a pronounced and steep absorption peak at approximately 2.0 μm (corresponding to an energy of 0.62 eV), which is typically identified as the first exciton peak (*34*, *35*).

Varying the infrared wavelength selectively accesses different transitions. At 2 μm wavelengths with low photon energy near the first excitonic peak, photons with energy just above the bandgap primarily excite the first excitonic transition from the highest heavy-hole state (1S_h_) to the lowest conduction state (1S_e_), producing a “cold” band-edge exciton (Process ①). At short wavelengths, photons with energy larger than the bandgap probably excite higher-energy transitions from the deeper 1P_h_ state to the same conduction state (1S_e_) due to the heavy-hole nature (Process ②). At even shorter wavelengths with even higher photon energy, more transition channels open up, including transitions to higher conduction states or from deeper valence bands (Processes ③ and ④). The simultaneous availability of multiple transition channels results in high absorption coefficients for infrared photons, which allows for the generation of high-density initial excitons. If the photon energy sufficiently exceeds the bandgap, the excess kinetic energy of the resulting hot carrier may probably be used to generate multiple electron-hole pairs from a single photon, rather than being entirely dissipated as heat via carrier cooling (*36*, *37*). Thus, different infrared wavelengths yield different numbers of initial excitons, with shorter wavelengths producing a higher initial exciton density due to the enhanced absorption from multiple transition channels and possible multiple exciton generation. Besides, the infrared intensity, by governing the photon flux, also directly determines the number of generated excitons. Given efficient charge extraction and transport within the photovoltaic HgTe CQDs/poly(3-hexylthiophene-2,5-diyl) (P3HT) heterojunction architecture, this higher exciton yield increases the quantity of photogenerated carriers delivered from the infrared detection unit to be transported to the light-emitting unit.

The light-emitting unit is designed with strategic energy alignment to route photogenerated holes into distinct color channels. It features a dual-emissive architecture, comprising a red-emitting layer (EML) with tris(1-phenylisoquinoline)iridium(III) (Ir(piq)_3_) doped in 4,4′-Bis(N-carbazolyl)-1,1′-biphenyl (CBP) and a cyan EML with bis[2-(4,6-difluorophenyl)pyridinato-C2,N](picolinato)iridium (Flrpic) doped in host CBP. A key design is the intentional 0.82 eV energy offset between their highest occupied molecular orbitals (HOMO: −5.24 eV for Ir(piq)_3_ and −6.06 eV for Flrpic, fig. S2), which creates a substantial hole-injection barrier between the two emitters. The structure of the overall integrated upconverter with the CQDs-based infrared detection and multicolor emission units is illustrated in Fig. 2D. Ultraviolet photoelectron spectroscopy measurements were used to provide a precise estimation of the band edge energy level (figs. S2 and S3), and the resulting energy band diagrams are shown in Fig. 2E, clarifying the operation mechanism of upconverters.

Under longer-wavelength or lower-intensity infrared illumination, the upconverter generates fewer excitons, resulting in a smaller number of photogenerated holes transported from the infrared detection unit to the multicolor emission unit. The photogenerated holes are transported through the hole transport layer (HTL) and preferentially injected and trapped into the red dopant Ir(piq)_3_ of the emission unit, owing to its shallower HOMO level relative to the host. The large hole-injection barrier between the red and cyan dopants confines holes within the red EML, while electrons migrate unimpeded through the electron transport layer (ETL), resulting in red emission. Conversely, shorter-wavelength or higher-intensity infrared illumination generates more excitons, thereby supplying a larger flux of photogenerated holes to the multicolor emission unit. This increased hole population can saturate the trap states in the red dopant. Subsequent holes then migrate via the HOMO level of the CBP host, overcome the energy barrier, and inject into the cyan EML, enabling simultaneous red and cyan emission. The emission color and luminance are determined by the total flux of photogenerated holes reaching the multicolor emission unit, which is jointly governed by the infrared wavelength and intensity. Control experiments utilizing a single EML with co-deposited red and cyan dopants within a shared host to equalize their physical distance from the infrared absorption layer confirm very few emitted color changes influenced by the infrared wavelength and intensity, proving the critical role of bilayer emitting architecture with the deliberate hole-injection barrier (fig. S4). To comprehensively elucidate the upconverter’s high performance and optimization process, we first detail the design and characterization of the HgTe CQDs-based infrared detection unit and the multicolor organic light-emitting unit separately, which are two key functional units. Following this, we present the integrated infrared-to-full-color upconversion performance to demonstrate the synergistic outcome.

### Infrared photodetection

The photovoltaic HgTe CQDs/ P3HT heterojunction PD units are employed in the upconverters to achieve superior infrared sensitivity and conversion efficiency of infrared photons to photogenerated carriers. The P3HT HTL constructs an efficient interfacial barrier that forms heterojunctions for HgTe CQDs to block dark current while enabling unimpeded photocarrier transport (fig. S5) (*38*). In addition, the existing tunneling current in the homojunction is also eliminated due to the absence of depletion regions in this heterojunction architecture (fig. S5) (*38*).

**Fig. 3.**
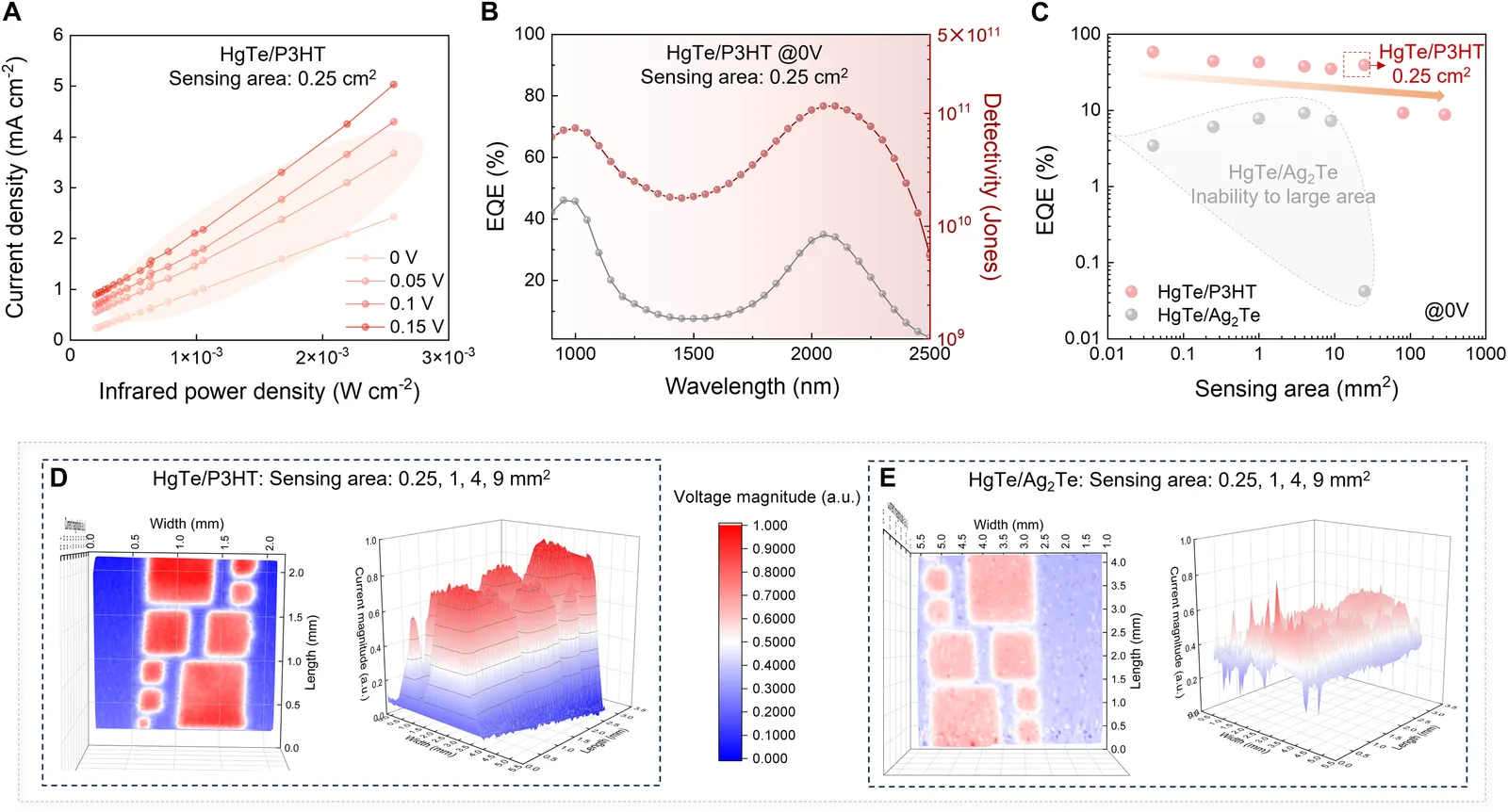
Infrared photodetection. (**A**) Current density versus infrared power density curves of the HgTe/P3HT PD for different bias voltages of 0, 0.05, 0.1, and 0.15 V with a calibrated 600°C blackbody as a light source. (**B**) *EQE* and detectivity versus wavelength curves of the HgTe/P3HT PD at 0 V. (**C**) *EQE* at 0 V calculated with a calibrated 600°C blackbody as a light source versus the sensing area of the HgTe/P3HT heterojunction and HgTe/Ag_2_Te homojunction. (**D**) Photocurrent mapping characteristics of the HgTe/P3HT heterojunction. (**E**) Photocurrent mapping characteristics of the HgTe/Ag_2_Te homojunction.

The current density-voltage curves of the HgTe/P3HT PD present typical rectification characteristics, as shown in fig. S6A. The dark current density of the HgTe/P3HT PD is low at 6.29 × 10^−3^ mA cm^−2^ and 8.53 × 10^−1^ mA cm^−2^ at applied voltages of 0 V and 0.2 V, respectively. At an infrared power of 2.57 × 10^−3^ W cm^−2^, the zero-bias photocurrent density reaches 2.42 mA cm^−2^, and the deduced current gain (defined as the ratio of the photocurrent density to the dark current density) is nearly 400 times greater. The HgTe/P3HT PD demonstrates that current density linearly increases with infrared intensity for different bias voltages (Fig. 3 and fig. S6A). The dependence of the open-circuit voltage (*V_OC_*) on the infrared intensity is shown in fig. S6B, with a maximum of 0.056 V. The variations in the external quantum efficiency (*EQE*, eq. S2) and specific detectivity (eq. S3) of the HgTe/P3HT PD with changes in the infrared wavelength are presented in Fig. 3B. The response spectrum of the HgTe/P3HT PD exhibits alternating peaks and troughs with a maximum *EQE* of 46% at a wavelength of 950 nm and a maximum detectivity of 1.15 × 10^11^ Jones at a wavelength of 2100 nm.

For HgTe CQD-based PD, photovoltaics are commonly achieved by a homojunction architecture through the introduction of silver telluride (Ag_2_Te) nanocrystals and the fixation of Ag^+^ ions within adjacent HgTe CQDs via p-type doping (*39*–*41*). However, the tunneling current in the depleted region of the homojunction photovoltaic detectors degrades the device performance (fig. S5B). In addition, the uncontrollable diffusion and spatially inhomogeneous doping process of homojunctions are unable to reach large sensing areas. Compared with the HgTe/P3HT heterojunction, the HgTe/Ag_2_Te homojunction shows much poorer device performance, with an *EQE* at 0 V that is only 0.2 times that of the heterojunction within the sensing areas lower than 9 mm^2^ (Fig. 3C). When the sensing area increases to 0.25 cm^2^, the *EQE* at 0 V of the HgTe/P3HT heterojunction is high at 39%, whereas that of the HgTe/Ag_2_Te homojunction is only 0.04%. With a further increase in the sensing area, the HgTe/Ag_2_Te homojunction shows no infrared response, and the *EQE* of the HgTe/P3HT heterojunction at 0 V decreases to a value similar to that of the homojunction at small sensing areas. To make the upconverter area as large as possible while ensuring detection and luminescence performance, the area of the HgTe/P3HT PD, OLED, and upconverter is chosen to be 0.25 cm^2^ to characterize the performance unless otherwise specified. In addition, the photocurrent maps of the HgTe/P3HT heterojunction and the HgTe/Ag_2_Te homojunction were measured, as shown in Fig. 3, D and E. The HgTe/P3HT heterojunction displays a much more uniform response distribution and a much superior signal-to-noise ratio at sensing areas of 0.25, 1, 4, and 9 mm^2^ compared with those of the HgTe/Ag_2_Te homojunction. The much-improved photoelectric conversion performance and film flatness of the HgTe/P3HT heterojunction PD unit are beneficial for further stacking the OLED unit to achieve high-performance, large-area upconverters.

### Multicolor emission

**Fig. 4.**
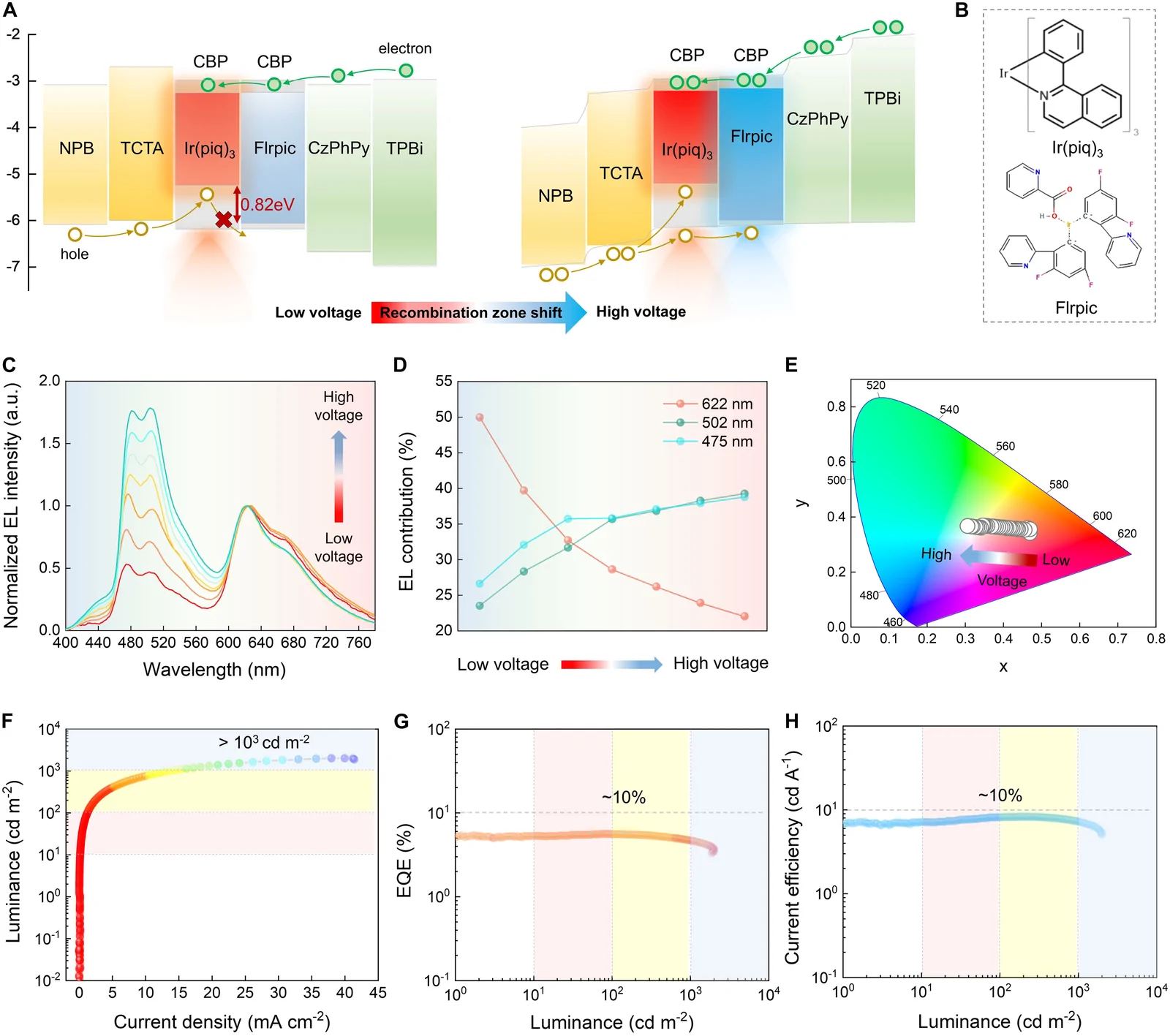
Multicolor emission. (**A**) Energy band diagram of the multicolor OLED. (**B**) Molecular structures of the red dopant Ir(piq)_3_ and the cyan dopant Flrpic. (**C**) Evolution of the normalized *EL* spectra of the multicolor OLED with increasing voltage. (**D**) *EL* contributions of the 622, 502, and 475 nm components to the entire *EL* spectrum of the multicolor OLED. (**E**) Evolution of the *CIE 1931* chromaticity coordinates of the multicolor OLED with increasing voltage. (**F**) Luminance versus current density curves of the multicolor OLED. (**G**) *EQE* versus luminance curves of the multicolor OLED. (**H**) Current efficiency versus luminance curves of the multicolor OLED. The area of the multicolor OLED is 0.25 cm^2^.

The energy barrier-modulated color emission for double-emissive-layer multicolor OLED units with elaborate hole-trapping energy level alignment is researched. The multicolor OLED is composed of indium-tin-oxide (ITO, electrode), HATCN (5 nm, hole injection layer, HIL), NPB (30 nm, HTL), TCTA (10 nm, HTL), 8% Ir(piq)_3_ in CBP (15 nm, red EML), 15% Flrpic in CBP (20 nm, cyan EML), CzPhPy (10 nm, ETL), TPBi (30 nm, ETL), and lithium fluoride/aluminum (LiF/Al, 1 nm/100 nm, electrode). As shown in Fig. 4A, at low driving voltage, holes are preferentially trapped by the red dopant Ir(piq)_3_, whose HOMO level (−5.24 eV) is substantially shallower than that of the CBP host (−6.18 eV). There is a large energy barrier of 0.82 eV between the HOMO of Ir(piq)_3_ red dopant (−5.24 eV) and the Flrpic cyan dopant (−6.06 eV) to prevent hole injection into the Flrpic cyan dopants, effectively confining holes within the red EML. Electrons transport freely from the cathode through the ETLs to the cyan and then the red dopant. Consequently, recombination occurs mainly in the red EML, yielding dominant red emission. As the voltage increases (Fig. 4A), the internal electric field strengthens, causing band-bending and a reduction of the energy barrier. More carriers are injected into the OLED. Once the limited trap sites of the red dopant are saturated, holes begin to enter the HOMO level of the CBP host in the red layer. Through transport between hosts, holes bypass the filled red-dopant traps and directly inject into the cyan EML. The recombination zone thus progressively shifts from the red to the cyan layer, leading to a continuous increase in the cyan emission component with rising voltage. Hole-only device measurements (fig. S7) corroborate this mechanism that the introduction of the Ir(piq)_3_ red-dopant strongly suppresses the low-bias current, confirming direct charge trapping on the Ir(piq)_3_ red-dopant (*42*). At high bias, the current-density-voltage slope of the doped device aligns with that of the pure-host device, indicating that traps are filled and switched to host-mediated transport (*42*). This bias-controlled transition from trap-limited to host-mediated hole transport enables continuously tunable multicolor emission in the OLED unit.

Flrpic has emission peak wavelengths of 475 and 502 nm, and Ir(piq)_3_ has an emission peak wavelength of 622 nm, as shown in the electroluminescence (*EL*) spectra (Fig. 4C). The strong voltage dependence of the *EL* spectral evolution and corresponding *EL* contributions of the 622, 502, and 475 nm components to the entire *EL* spectrum of the multicolor OLED are shown in Fig. 4, C and D, respectively. When the voltage is low, the intensity of the 622 nm *EL* peak is much greater than that of the 502 and 475 nm peaks. With increasing voltage, the intensities of the 502 and 475 nm *EL* peaks gradually increase and become stronger than those of the 622 nm peak. As a result, the *CIE 1931* chromaticity coordinates of the multicolor OLED evolve with voltage and are (0.47, 0.34) at low bias and (0.30, 0.37) at high bias, as shown in Fig. 4E. A similar trend of voltage-controlled color changes is also demonstrated in the *CIE 1976* chromaticity coordinates of the multicolor OLED, as presented in fig. S8. The optimization process of the host material and the thicknesses of the red and cyan EML have been explored, as shown in figs. S9 to S12.

In addition to the large color-tunable regions of the multicolor OLED, the luminance and efficiency are also superior. The maximum luminance of the multicolor OLED is high at 2003.78 cd m^−2^ at a current density of 39.97 mA cm^−2^, as shown in Fig. 4F. In addition, the multicolor OLED possesses an *EQE* value (eq. S4) of nearly 6% and a current efficiency of nearly 9 cd A^−1^ over the entire range of luminance, showing excellent injected electrons-to-emitted photons conversion ability (Fig. 4, G and H).

### Infrared-to-full-color upconversion

**Fig. 5.**
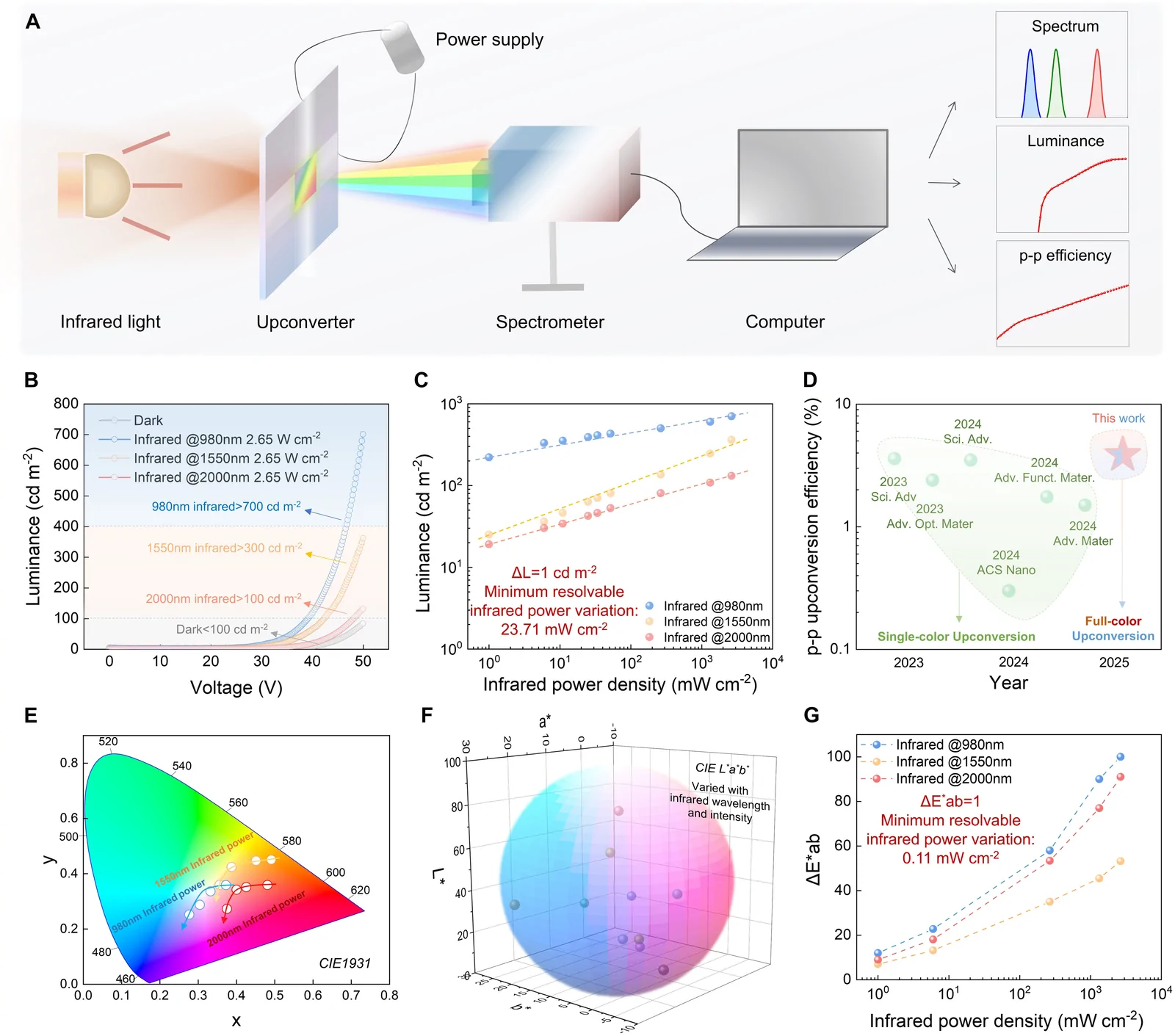
Infrared-to-full-color upconversion. (**A**) Schematic diagram of the measurement process for the upconverter. (**B**) Luminance versus applied voltage curves of the upconverter without (dark) and with infrared illumination at different wavelengths of 980, 1550, and 2000 nm under the same intensity of 2.65 W cm^−2^. (**C**) Luminance versus infrared power density curves of the upconverter with infrared illumination at different wavelengths of 980, 1550, and 2000 nm. (**D**) The *p-p* upconversion efficiency of the infrared-to-full-color upconverter compared with the reported representative single-color upconverters. (**E**) Evolution of the *CIE 1931* chromaticity coordinates of the upconverter with increasing infrared power density for different infrared wavelengths of 980, 1550, and 2000 nm. (**F**) Evolution of the CIE L^*^a^*^b^*^ color space of the upconverter with various infrared wavelengths and intensities. (**G**) Emitted visible light color difference versus infrared power density of the upconverter at different infrared wavelengths of 980, 1550, and 2000 nm. The area of the full-color upconverter is 0.25 cm^2^.

Leveraging the engineering of the quantum-confined states in the detection unit and the dual-emissive layer together with the hole-injection barrier in the emitting unit, the infrared-to-visible upconversion with correlated wavelength/intensity- color/luminance mapping is demonstrated. A visible transparent electrode of 2 nm Al/8 nm Ag/30 nm WO_3_ was used as the top electrode, and an infrared transparent electrode of ITO was used as the bottom electrode. The upconverter operates in transmissive mode with bottom-illuminated top emission, which is more practical and efficient than reflective mode with an opaque top electrode (*43*). The performance of the semitransparent infrared-to-full-color upconverter was characterized by the measurement setup presented in Fig. 5A.

The luminance versus applied voltage curves of the upconverter without and with infrared illumination at different wavelengths of 980, 1550, and 2000 nm are shown in Fig. 5B. In the absence of infrared light, the upconverter could hardly emit bright visible light with a luminance below 100 cd m^−2^ even under a high driving voltage. Under infrared illumination, the upconverter turns on with the shorter infrared wavelengths exhibiting a smaller turn-on voltage. The turn-on voltage of the upconverter is higher than that of the multicolor OLED, maybe due to the additional interfacial barriers and voltage losses inherent in the integrated structure (*28*). The luminance of the upconverter under 980 nm infrared illumination is above 700 cd m^−2^, which is almost twice that under 1550 nm infrared irradiation and five times that under 2000 nm infrared stimulation with the same intensities. Although the shorter wavelengths have a lower photon flux at the same optical power, the higher photon energy probably facilitates multiple exciton generation and accesses multi-excitonic transition channels within the quantum-confined HgTe CQDs, leading to a greater initial exciton yield and subsequently a higher density of photogenerated carriers. These additional carriers can overcome the hole-injection barrier between the red and cyan emissive layers, simultaneously activating both layers and resulting in a mixed visible emission with higher luminance. With respect to the different infrared wavelengths, the luminance of the upconverter is nearly linearly related to the infrared power density, with stretching more than three orders of magnitude from 1 to 10^3^ mW cm^−2^, as presented in Fig. 5C. Near a luminance level of ∼100 cd m^−2^, a just-noticeable difference in luminance for the human eye is approximately 1 cd m^−2^ (*44*). Based on this criterion, we determined that a minimum infrared power density increment of 23.71 mW cm^−2^ is required to induce a perceptible change in visible brightness. In addition, the full-color upconverter has a high infrared photon-to-visible photon (*p-p*) upconversion efficiency of 3.85% (eq. S5), which is comparable to that of previously reported single-color upconverters (Fig. 5D).

The trajectories of the *CIE 1931* (Fig. 5E) and *1976* (fig. S13) chromaticity coordinates of the emission-visible colors of the upconverter evolve with increasing infrared power density under different infrared wavelengths of 980, 1550, and 2000 nm illumination. With increasing infrared intensities, the *CIE 1931* chromaticity coordinates change from (0.372, 0.359) to (0.276, 0.252) under 980 nm infrared stimulation, from (0.491, 0.451) to (0.355, 0.360) under 1550 nm infrared illumination, and from (0.481, 0.359) to (0.375, 0.273) with 2000 nm infrared stimulation. The sensitive infrared wavelength/intensity-dependent visible light color/ luminance is displayed in the CIE L^*^a^*^b^*^ color space with L^*^ for perceptual lightness and a^*^ and b^*^ for the four unique colors of human vision: red, green, blue, and yellow (Fig. 5F). The combination of color and luminance provides a higher-dimensional encoding space for discriminating infrared wavelength and intensity. The color difference ΔE^*^_ab_ calculated by eq. S6 conforms to the ability of the human eye to perceive color differences while considering both luminance and color. As the infrared power density increases, the color difference in the visible light emitted by the upconverter increases, as displayed in Fig. 5G. Different infrared wavelengths of 980, 1550, and 2000 nm induce diverse color differences. Specifically, by defining a color difference of ΔE^*^_ab_ = 1 as the just-noticeable difference threshold, the corresponding required infrared power density increments for a human to discern a color-luminance combined change can be determined as low as 0.11 mW cm^−2^. This is nearly 200-fold smaller than the 23.71 mW cm^−2^ required to distinguish a pure luminance variation (Fig. 5C), with sensitivity significantly exceeding two orders of magnitude. It accords with the ability of the human eye more sensitive to color differences than to brightness changes alone. The superior chromatic sensitivity of this infrared-to-full-color upconverter significantly enhances the discrimination and identification of subtle infrared fluctuations for high-fidelity visualization, far surpassing traditional single-color modes that rely solely on luminance contrast.

### Infrared visualization wearable eyeglass

In the infrared-to-full-color upconverter, the carriers tend toward vertical transport across multiple functional layers with the applied electric field, allowing high-resolution pixel-free infrared wavelength and intensity-tunable full-color imaging to be visualized by human eyes. A lightweight (23 g upconverter with encapsulation in total) infrared visualization wearable eyeglass is demonstrated. The differences in the absorption of infrared light by different substances from imaging objects lead to changes in the wavelength and intensity of the reflected infrared light, which is detected by the infrared-to-full-color upconverter, and emits the corresponding varied visible colors and luminance object-shaped patterns with the infrared signature to project onto the retina.

**Fig. 6.**
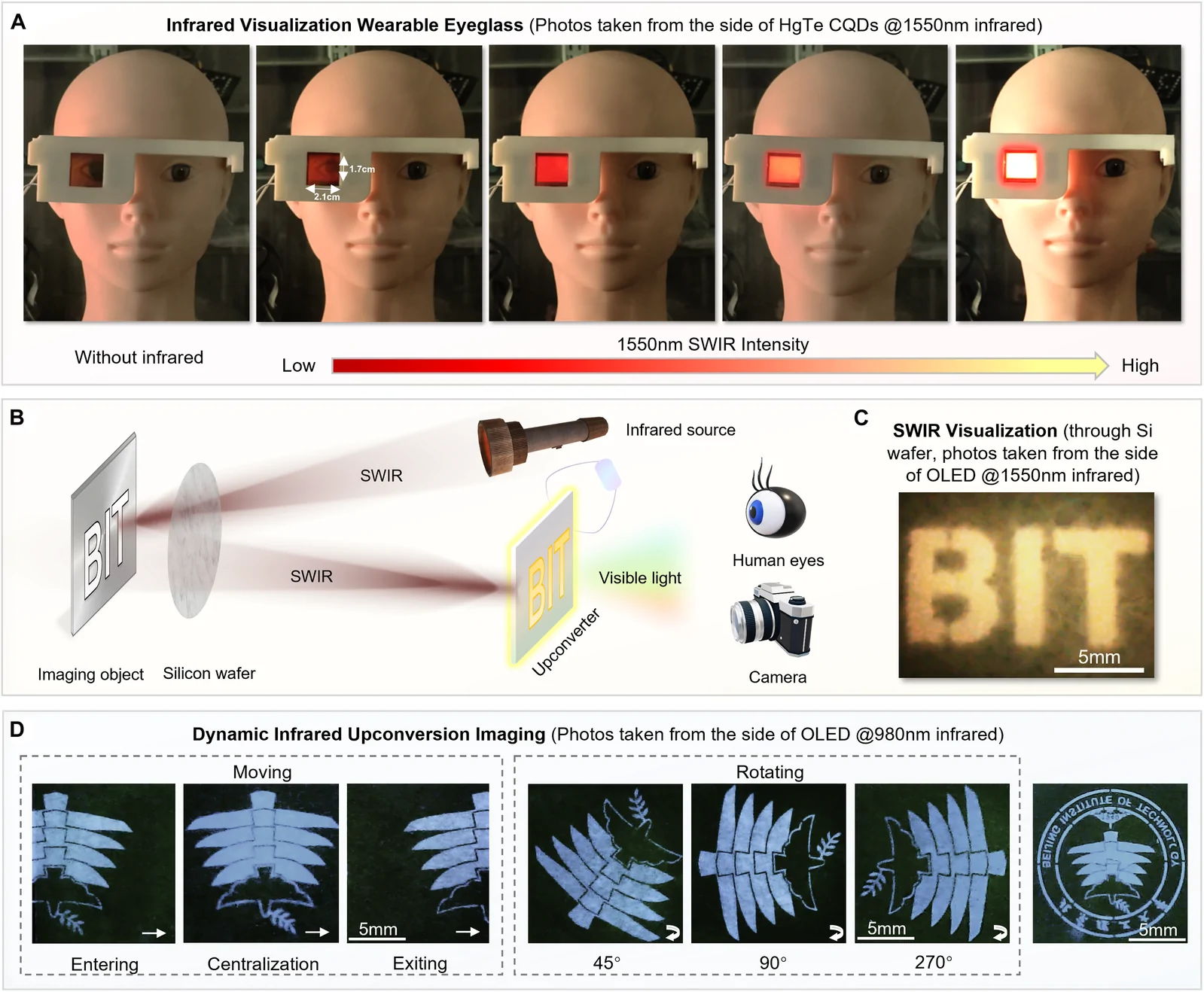
Infrared visualization wearable eyeglass. (**A**) Infrared visualization wearable eyeglass displaying infrared intensity-controlled emitted color and luminance variation. (**B**) Schematic of the imaging process of the object behind a silicon wafer. (**C**) SWIR visualization demonstration when the mask is covered by a silicon wafer. (**D**) Dynamic infrared upconversion imaging when the object is moving (entering, centralizing, exiting) with the speed of 1 cm s^−1^ or rotating (45°, 90°, 270°) with the speed of 0.25 rad s^−1^. The infrared wavelengths are [(A) and (C)] 1550 nm and (D) 980 nm. The photos were taken from the (A) bottom electrode and [(C) and (D)] top electrode of the upconverter. The area of the full-color upconverter is 3.57 cm^2^ for imaging demonstration.

The upconverter possesses wearability and infrared visualization ability in actual usage, as shown in Fig. 6A. The upconverter generates clear and uniform luminance even with a large device area of 3.57 cm^2^ under 1550 nm SWIR stimulation. Because the naked eye observes the image from the transparent top electrode of the upconverter and the displayed photos were taken from the bottom brownish HgTe CQDs, the photographed colors of the upconverters (bottom side) are redder than those observed by the eye (top side). As the incident 1550 nm SWIR intensity increases, the upconverter successively displays uniform large areas of emission of deep red, red, orange, and yellow colors with increased luminance, demonstrating the infrared intensity-tunable full-color visualization capacity. Interestingly, the upconverter could interact with users to identify the intensity of invisible infrared power from the naked-eye perceivable visible colors, with low-brightness red representing faint infrared irradiation and bright-luminous yellow representing stronger infrared power. In addition, the infrared visualization wearable eyeglass enables the unveiling and visualization of invisible intrusive infrared signals that threaten our privacy in our daily lives, realizing anti-detection and countersurveillance functions. The semi-transparent nature of the upconverter prototype naturally results in the superposition of the infrared upconverted visible signal and the original visible background. This dual-vision characteristic offers flexible operational modes for different application scenarios. For instance, in a virtual reality (VR)-like or pure infrared imaging mode, the visible background can be easily eliminated by integrating a visible-light-cutoff filter, ensuring a high-contrast infrared-only display. Conversely, for augmented reality (AR) applications (*45*), the semi-transparency is a significant advantage, as it allows users to perceive the physical environment while simultaneously receiving multispectral infrared information. This versatile integration potential suggests that our upconverter can serve as a core component for the next generation of intelligent wearable vision systems, providing a seamless transition between reality and augmented infrared information.

In addition, the silicon wafer is located between the SWIR light source and the imaging object to demonstrate the SWIR visualization ability of the upconverter, as shown in Fig. 6B. The silicon wafer is opaque in the visible region but allows significant transmission for 1550 nm SWIR illumination. High-quality “BIT”-shaped images of the upconverter are clearly observed from the top electrode with bright yellow luminance, even though the mask is covered by a silicon wafer (Fig. 6C). Furthermore, the upconverter displays dynamic infrared upconversion imaging by moving (entering, centralizing, exiting) and rotating (45°, 90°, 270°) the object (Fig. 6D). The dynamic infrared upconversion imaging with various motion velocities is shown in fig. S14. The pattern of the school emblem “Beijing Institute of Technology” with clear-cut boundaries, high contrast, and high resolution is shown on the upconverter under the 980 nm infrared stimulus. The emitted color of the upconverter when the infrared wavelength is 980 nm exhibits a different color when the illuminated infrared wavelength is 1550 nm, which is consistent with the evolution of the *CIE* with the infrared wavelength shown in Fig. 5E. As a result, the upconverter demonstrates the strong dependence of the emitted color and luminance on the incident infrared wavelength and intensity, enabling high-resolution and pixel-free infrared-to-full-color upconversion imaging.

### Infrared perception retinal photoreceptor

**Fig. 7.**
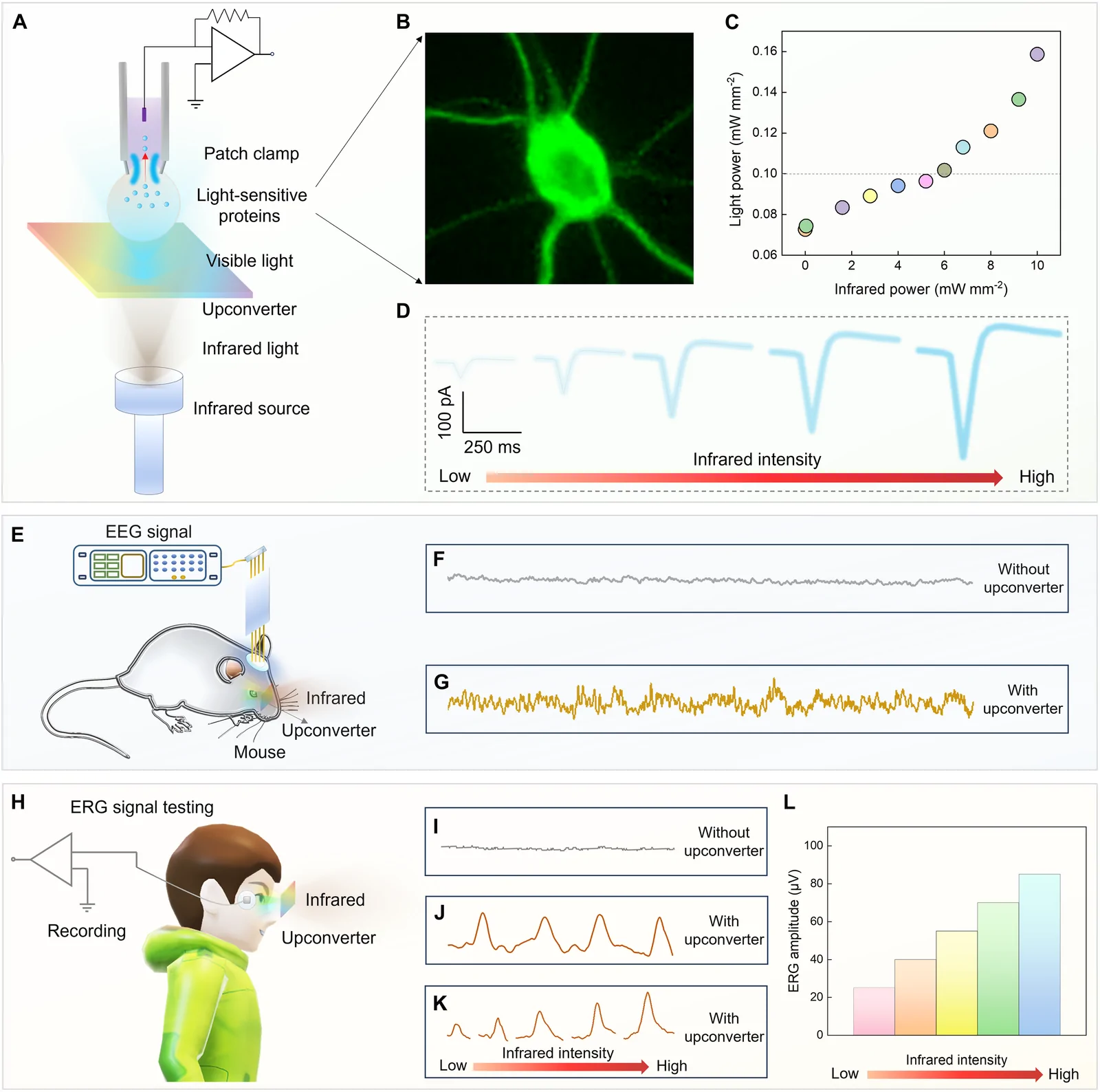
Infrared perception retinal photoreceptor. (**A**) Schematic illustration of the setup for stimulating light-sensitive proteins bound to upconverters under infrared illumination. (**B**) Representative fluorescence image of ChR2-expressing neurons. (**C**) Light power versus infrared power curves of the single-color upconverter. (**D**) Recordings of photocurrents in ChR2 neurons in response to pulsed infrared illumination at different intensities. (**E**) Schematic illustration of testing the EEG signal of the mice. (**F**) EEG response traces under infrared illumination of the mice without the upconverter. (**G**) EEG response traces under infrared illumination of the mice with the upconverter. (**H**) Schematic illustration of testing the ERG signal of humans. (**I**) ERG response traces under infrared illumination of humans without the upconverter. (**J**) ERG response traces under infrared illumination of humans with the upconverter. (**K**) ERG response traces under infrared illumination of humans with the upconverter at increasing infrared intensities. (**L**) ERG amplitudes of the light photovoltage with the upconverter at increasing infrared intensities.

To establish infrared vision at the neural level, we engineered the upconverter as an implantable bionic photoreceptor through integration with channelrhodopsin-2 (ChR2), a widely adopted blue-light-sensitive opsin (*46*–*48*). As shown in Fig. 7A, the upconverter is positioned beneath ChR2-expressing neurons and illuminated with the infrared light. The fluorescence image of the ChR2-expressing neurons is displayed in Fig. 7B. Under 980 nm infrared illumination (10 mW mm^−2^), the upconverter generates blue light with a peak spectrum around 470 nm, delivering the light power of 0.16 mW mm^−2^ at the cell interface, surpassing the activation threshold of 0.1 mW mm^−2^ for ChR2 (Fig. 7C). The whole-cell patch-clamping was performed to record the intracellular signals under infrared illumination (Fig. 7D). The amplitude of induced photocurrents is enhanced with increasing infrared intensities. Behaviors of light-sensitive proteins bound to upconverters when excitation at 1550 nm or 2000 nm are shown in section S6. The results demonstrate that the light-sensitive proteins bound to upconverters can be reliably activated by infrared illumination, generating photocurrents that may possibly stimulate retinal neurons to enable infrared vision, even to potentially bypass damaged photoreceptor cells to restore visual function.

We further recorded electroencephalography (EEG) signals in mice with and without upconverters under infrared illumination (Fig. 7E). In the absence of the upconverter, infrared pulses elicited negligible responses (Fig. 7F). In contrast, with the upconverters, the infrared pulses generate robust EEG signals, indicating infrared-driven neural synchronization, as shown in Fig. 7G. Then, we proceed to validate whether the infrared light could effectively activate the visual systems of humans through the upconverters. Electroretinography (ERG) of humans, as an electrophysiological signal in the visual system, was recorded under infrared illumination (Fig. 7H). Figure 7I demonstrates that infrared light could not evoke any ERG signals in humans without upconverters. However, integration of the upconverter with human eyes generates synchronous ERG traces upon infrared light illumination to the eye (Fig. 7J). ERG signal amplitude scales increase with upconverter emission brightness stimulated with increasing infrared power, as shown in Fig. 7K. The corresponding ERG amplitudes of the light photovoltage with the upconverter at increasing infrared intensities are displayed in Fig. 7L. These findings confirm that the upconverter enables functional infrared perception in the human visual system, supporting its potential as both a retinal prosthesis and a sensory expansion.

## DISCUSSION

We redefine infrared vision by transcending the monochrome paradigm, translating infrared spectral and intensity signatures into discernible color variations rather than mere brightness changes. By harnessing the human eye’s superior sensitivity to chromatic variation, our HgTe CQD-based full-color upconverter achieves a discrimination sensitivity for subtle infrared variations exceeding more than two orders of magnitude higher than conventional single-color modes while maintaining high luminance (>700 cd m^−2^) visible emission and the upconversion efficiency of 3.85%. The photon-energy-selective excitonic transitions in the quantum-confined states govern the wavelength-dependent exciton yield, and the incident photon flux correlated by infrared intensity determines the number of excitons generated. This functionality is coupled with a dual-emissive OLED architecture featuring an engineered hole-injection barrier, enabling color routing that directly correlates with the number of photogenerated carriers. We demonstrate two transformative application routes: a lightweight, semi-transparent wearable eyeglass that projects multispectral infrared as full-color vision directly onto the retina, and potentially a bio-integrated implantable retinal photoreceptor by binding upconverters to light-sensitive proteins, enabling innate infrared perception. By surpassing the evolutionary boundaries of biological photoreception, this technology paves the way for next-generation visual prosthetics and human-integrated sensory expansion, high-fidelity augmented reality, molecularly sensitive substance identification, and robust navigation in degraded visual environments.

## MATERIALS AND METHODS

### Materials

Dipyrazino[2,3-f:2″,3″-h] quinoxaline-2,3,6,7,10,11-hexacarbonitrile (HATCN), N,N“-bis(naphthalen1-yl)-N,N”-bis(phenyl) benzidine (NPB), tris(1-phenylisoquinoline)iridium(III) (Ir(piq)_3_), 4,4′-Bis(N-carbazolyl)-1,1′-biphenyl (CBP), bis[2-(4,6-difluorophenyl)pyridinato-C2,N](picolinato)iridium (Flrpic), 4,6-bis(3-(9H-carbazol-9-yl)phenyl)pyrimidine (CzPhPy), and 2,2′,2″-(1,3,5-Benzinetriyl)-tris(1-phenyl-1-H-benzimidazole) (TPBi) were purchased from the Tansoole chemical procurement platform. 4,4″,4″“-Tris(carbazol-9-yl) triphenylamine (TCTA) was purchased from Xi’an p-OLED Company.

### Synthesis of HgTe CQDs

The synthesis of HgTe CQDs was similar to that in previous reports (*49*). Mercury chloride (HgCl_2_, Strem Chemicals, 99%, 0.4 mmol) was dissolved in 16 ml of oleylamine (OAM, Aladdin) in a 40 ml glass vial at 100°C for 1 hour with stirring in the glove box. The temperature was then adjusted to the reaction temperature and stabilized for 30 min. Tellurium powder (Te, Sigma-Aldrich, 99.999%) in a trioctylphosphine (TOP, Sigma-Aldrich, 97%) solution (1 M, 0.4 ml) was rapidly injected. The clear solution immediately turned black. The reaction temperature and time are changed according to the target CQD size. The reaction was quenched by injecting a solution of 3.6 ml of dodecanethiol (DDT; Sigma-Aldrich, 98%) and 1.2 ml of TOP in 16 ml of tetrachloroethylene (TCE; Aladdin, 98%). After quenching, the vial was quickly removed from the glove box and cooled. The solution was precipitated with an equal volume of isopropanol (IPA) and centrifuged at 6000 rpm for 3 min. Finally, the precipitate was resuspended in 16 ml of chlorobenzene (CBZ, Aladdin, 99.5%) and stored under ambient conditions.

The absorption spectra of the HgTe CQD materials were characterized by a Fourier transform spectrometer (Nicolet iS20 FTIR Spectrometer). Transmission electron microscopy (TEM) images of the HgTe CQD materials were obtained via an FEI Talos F200x TEM instrument.

### Fabrication and characterization of HgTe CQD-based PD units

The ITO layer was deposited on the substrate using magnetron sputtering coating equipment. Before spin-coating the CQD solutions, the substrate was treated with 3-mercaptopropyltrimethoxysilane (MPTS) for 30 s and rinsed with IPA. The HgTe CQD solution was spin-coated on the substrate to construct films. Each HgTe CQD layer was exposed to a 10 mM HgCl_2_ methanol solution for 10 s, rinsed with IPA, crosslinked with a 1,2-ethanedithiol (EDT)/hydrochloric acid (HCl)/IPA (1:1:200 by volume) solution for 10 s, rinsed with IPA, and dried. The number of layers was determined by the required film thickness. The poly(3-hexylthiophene-2,5-diyl) (P3HT, 10 mg ml^−1^) was spin-coated on the HgTe CQD layers. Finally, molybdenum trioxide (MoO_3_, 3 nm) and silver (Ag) electrodes (50 nm) were deposited by thermal evaporation.

The calibrated 600°C blackbody was used as the infrared light source to investigate the performance. Current versus voltage curves were measured via a source meter (Keithley 2602B). Photocurrent mapping measurements using a laser light source and a point-by-point scan through an x-y translation stage were used to obtain the photocurrent response at every point of the measured area of the photodetection unit.

### Fabrication and characterization of multicolor OLED units

The substrate was sequentially cleaned in deionized water, acetone, ethanol, and IPA through an ultrasonic process and then treated with plasma for 15 min. The ITO layer was deposited on the substrate using magnetron sputtering coating equipment. The layers of HATCN (5 nm), NPB (30 nm), TCTA (10 nm), 8% Ir(piq)_3_ in CBP (15 nm), 15% Flrpic in CBP (20 nm), CzPhPy (10 nm), TPBi (30 nm), and LiF/Al (1 nm/100 nm) were subsequently thermally evaporated on the ITO layer under high vacuum (2 × 10^−6^ Torr).

#### Control experiment of optimizing EML host materials

The layers of HATCN (5 nm), NPB (30 nm), TCTA (10 nm), 8% Ir(piq)_3_ in TCTA (15 nm), 15% Flrpic in CzPhPy (20 nm), CzPhPy (10 nm), TPBi (30 nm), and LiF/Al (1 nm/100 nm) were subsequently thermally evaporated on the ITO layer under high vacuum (2 × 10^−6^ Torr).

The layers of HATCN (5 nm), NPB (30 nm), TCTA (10 nm), 8% Ir(piq)_3_ in CBP (15 nm), 15% Flrpic in CzPhPy (20 nm), CzPhPy (10 nm), TPBi (30 nm), and LiF/Al (1 nm/100 nm) were subsequently thermally evaporated on the ITO layer under high vacuum (2 × 10^−6^ Torr).

#### Control experiment of optimizing EML thickness

The layers of HATCN (5 nm), NPB (30 nm), TCTA (10 nm), 8% Ir(piq)_3_ in CBP (5 nm), 15% Flrpic in CBP (20 nm), CzPhPy (10 nm), TPBi (30 nm), and LiF/Al (1 nm/100 nm) were subsequently thermally evaporated on the ITO layer under high vacuum (2 × 10^−6^ Torr).

The layers of HATCN (5 nm), NPB (30 nm), TCTA (10 nm), 8% Ir(piq)_3_ in CBP (15 nm), 15% Flrpic in CBP (10 nm), CzPhPy (10 nm), TPBi (30 nm), and LiF/Al (1 nm/100 nm) were subsequently thermally evaporated on the ITO layer under high vacuum (2 × 10^−6^ Torr).

#### Control experiment of hole-only devices

ITO, HATCN (5 nm), NPB (30 nm), TCTA (10 nm), 8% Ir(piq)_3_ in CBP (15 nm), NPB (10 nm), Al (150 nm), and ITO, HATCN (5 nm), NPB (30 nm), TCTA (10 nm), CBP (15 nm), NPB (10 nm), Al (150 nm).

The electroluminescence (*EL*) spectra, luminance, and efficiency of visible light emitted by the OLEDs were measured by a spectrometer (XQEPRO-EL, Ocean Insight) with an integration sphere (FOIS-1, Ocean Insight).

### Fabrication and characterization of infrared-to-full-color visible upconverters

The multicolor emission units of NPB (30 nm), TCTA (10 nm), 8% Ir(piq)_3_ in CBP (15 nm), 15% Flrpic in CBP (20 nm), CzPhPy (10 nm), TPBi (30 nm), and LiF (1 nm) were stacked on the photodetection unit of HgTe/P3HT/MoO_3_ (3 nm). The bottom electrode was ITO, and the top electrode was Al (2 nm)/Ag (8 nm)/WO_3_ (30 nm).

Control experiment of co-deposit red and cyan dopants within a shared host: The multicolor emission units of NPB (30 nm), TCTA (10 nm), 8% Ir(piq)_3_ and 15% Flrpic in CBP (15 nm), CzPhPy (10 nm), TPBi (30 nm), and LiF (1 nm) were stacked on the photodetection unit of HgTe/P3HT/MoO_3_ (3 nm). The bottom electrode was ITO, and the top electrode was Al (2 nm)/Ag (8 nm)/WO_3_ (30 nm).

The ultraviolet photoelectron spectroscopy measurements were performed using Thermo Fisher ESCALAB 250Xi+. The spectra, luminance, and efficiency of visible light emitted by the upconverters were measured by a spectrometer (XQEPRO-EL, Ocean Insight) with an integration sphere (FOIS-1, Ocean Insight). During the imaging process, the maximum distance between the light source and the device is generally around 1 m.

### Bioexperiment of infrared-to-full-color upconverters

Electrophysiological properties of the neurons were measured with the whole-cell patch-clamp recording technique. The data were acquired and analyzed using Clampfit 10.3 software. The fluorescence image was captured using a fluorescence imaging microscope. All animal procedures were performed by Shanghai Sanzhuowu Technology Co., Ltd. using our offered upconverters on the basis of the guidelines of the National Institutes of Health for the care.

## Supplementary Materials

### This PDF file includes:

Sections S1 to S6 Figs. S1 to S15 References
