# Supplementary material for: Multispectral infrared-to-full-color upconversion expanding human vision
Source: Sci Adv. 2026 Jul 29;12(31):eaed0245. doi: 10.1126/sciadv.aed0245 (PMC13418540; doi:10.1126/sciadv.aed0245)
Supplement: Supplementary file 1 [file sciadv.aed0245_sm.pdf]

Science Advances
AAAS

Supplementary Materials for

Multispectral infrared-to-full-color upconversion expanding human vision

Chengchang Fu *et al.*

Corresponding author: Xin Tang, xintang@bit.edu.cn; Ge Mu, gemu@bit.edu.cn

Sci. Adv. **12**, eaed0245 (2026)
DOI: 10.1126/sciadv.aed0245

This PDF file includes:

Sections S1 to S6
Figs. S1 to S15
References

Section S1. Design Rationale and Operational Mechanism

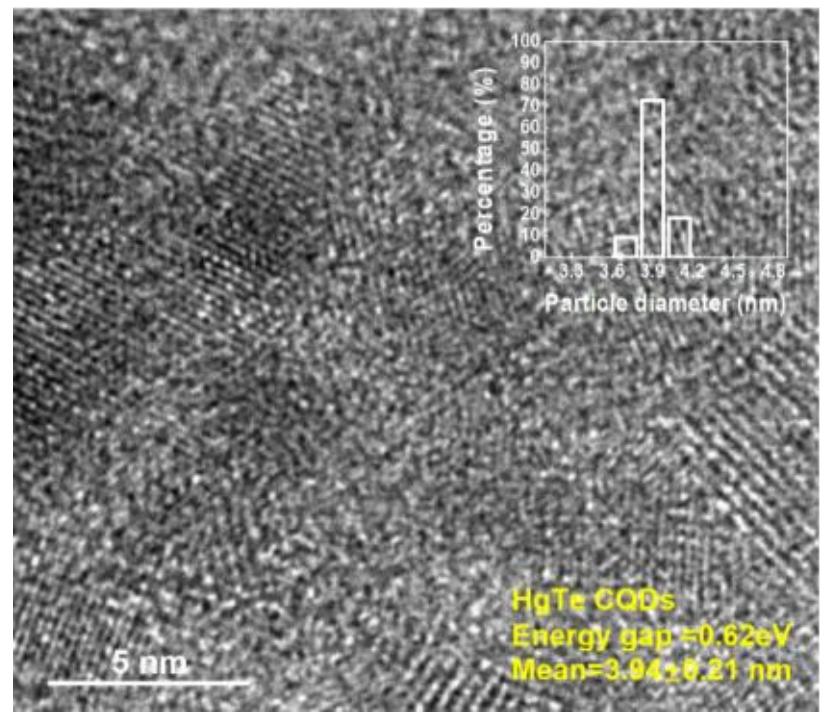

Fig. S1. Material Characterization.

The transmission electron microscope (TEM) image and size distribution of synthesized mercury telluride (HgTe) colloidal quantum dots (CQDs) with an energy gap of 0.62 eV and a mean size of 3.94±0.21 nm.

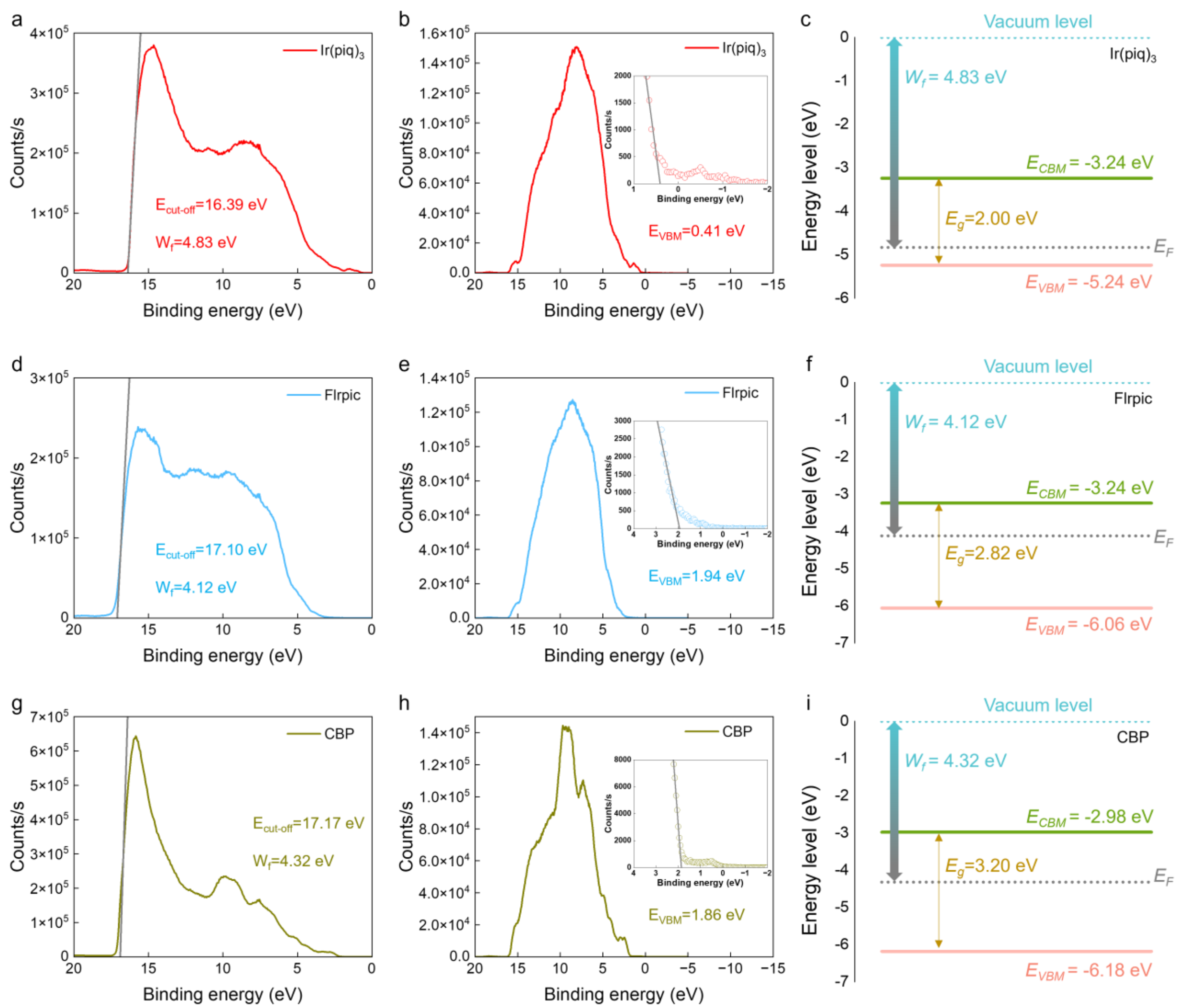

Fig. S2. Energy Band Characterization.

The ultraviolet photoelectron spectroscopy (UPS) spectra and the determination of the workfunction and valence band positions, and the energy band diagram for **(a-c)** tris(1-phenylisoquinoline)iridium(III) ($Ir(piq)_3$), **(d-f)** bis[2-(4,6-difluorophenyl)pyridinato-C2,N](picolinato)iridium (Flrpic), and **(g-i)** 4,4′-Bis(N-carbazolyl)-1,1′-biphenyl (CBP). The vacuum level is set to zero energy. The E_F, W_f, E_g, E_{CBM}, and E_{VBM} correspond to Fermi level, workfunction, energy bandgap, conduction band minimum, and valence band maximum, respectively.

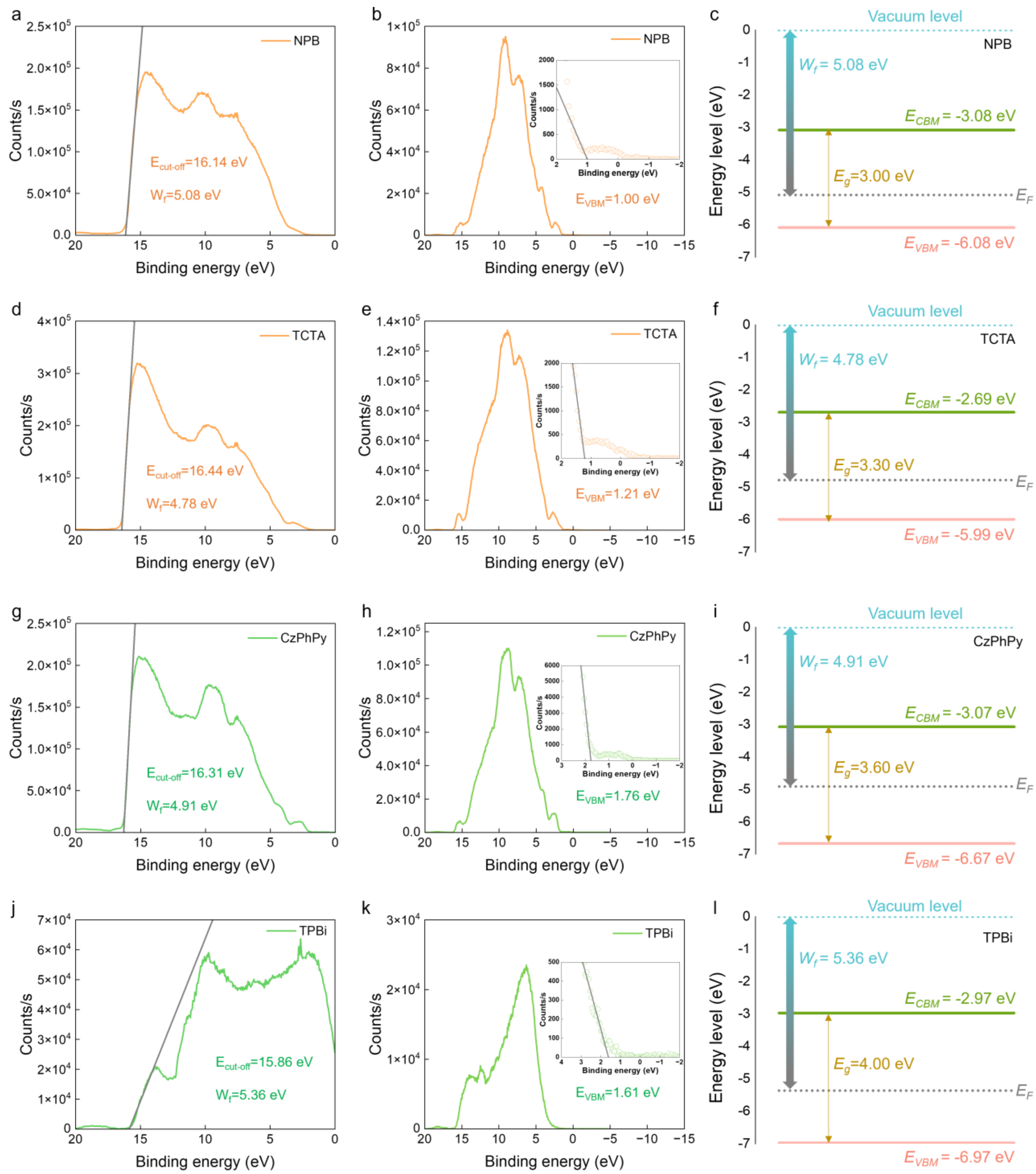

Fig. S3. Energy Band Characterization.

The UPS spectra and the determination of the workfunction and valence band positions, and the energy band diagram for (**a-c**) N,N"-bis(naphthalen1-yl)-N,N"-bis(phenyl) benzidine (NPB), (**d-f**) 4,4",4""-Tris(carbazol-9-yl) triphenylamine (TCTA), (**g-i**) 4,6-bis(3-(9H-carbazol-9-yl)phenyl)pyrimidine (CzPhPy) and (**j-l**) 2,2',2"-(1,3,5-Benzinetriyl)-tris(1-phenyl-1-H-benzimidazole) (TPBi). The vacuum level is set to zero energy. The E_F, W_f, E_g, E_{CBM}, and E_{VBM} correspond to Fermi level, workfunction, energy bandgap, conduction band minimum, and valence band maximum, respectively.

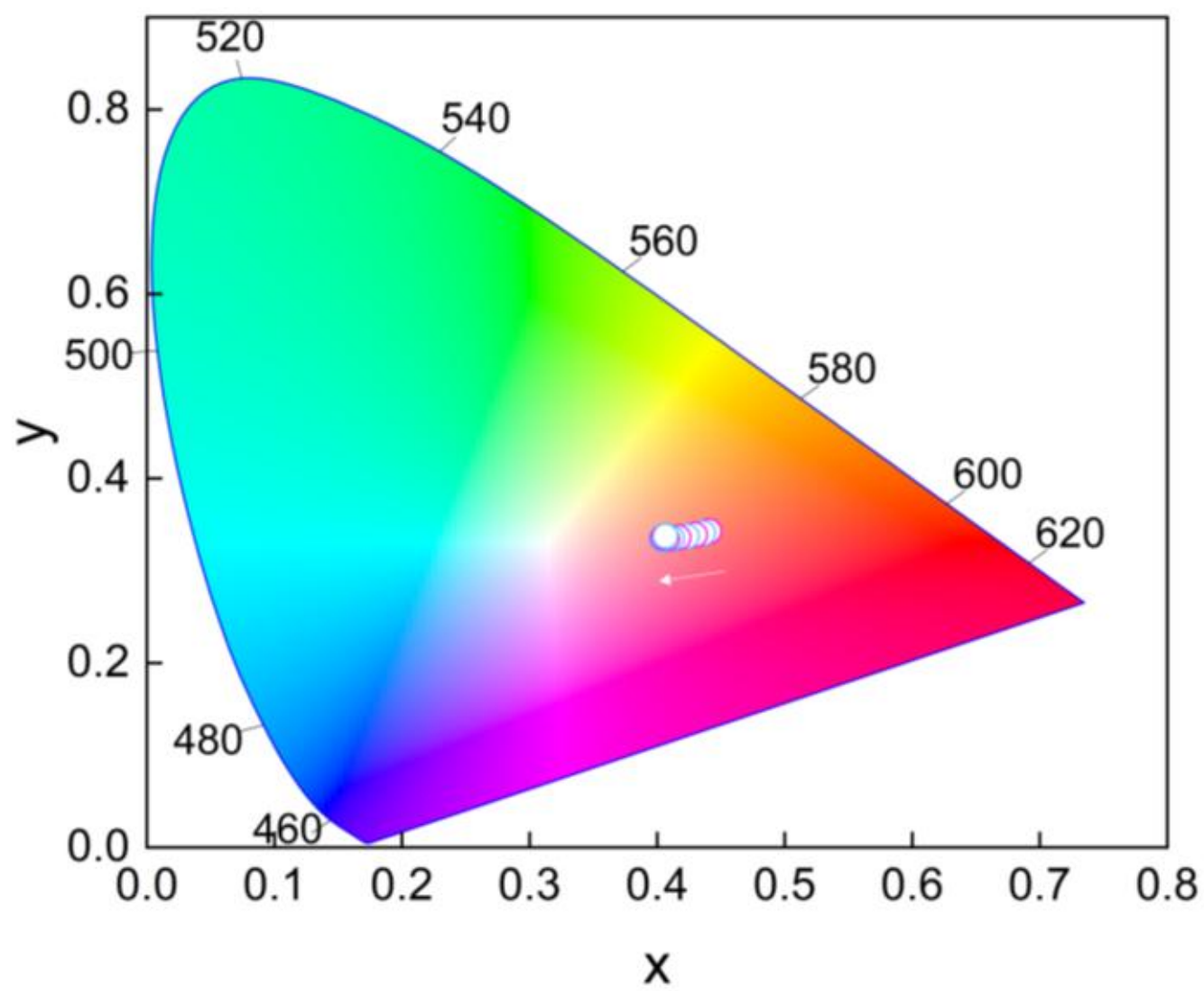

Fig. S4. Control Experiment.

The control experiment for the light-emitting layer co-deposited with red and cyan dopants within a common host. *CIE 1931* chromaticity coordinates measured under different infrared wavelengths (980 nm: blue circles; 2000 nm: pink circles) and intensities. The data points from right to left correspond to increasing infrared power densities of 1, 6, 265.39, 1326.96, and 2653.92 mW cm^{-2} for each wavelength. The arrow indicates the direction of increasing intensity. In this control device, the slight color shift arises probably because photogenerated holes preferentially occupy the shallower red dopant at low carrier flux, while at much higher flux, partial hole transfer to the cyan dopant occurs. However, this color shift is quantitatively small compared with that of the bilayer emissive architecture featuring a designed hole-injection barrier.

Section S2. Infrared Photodetection

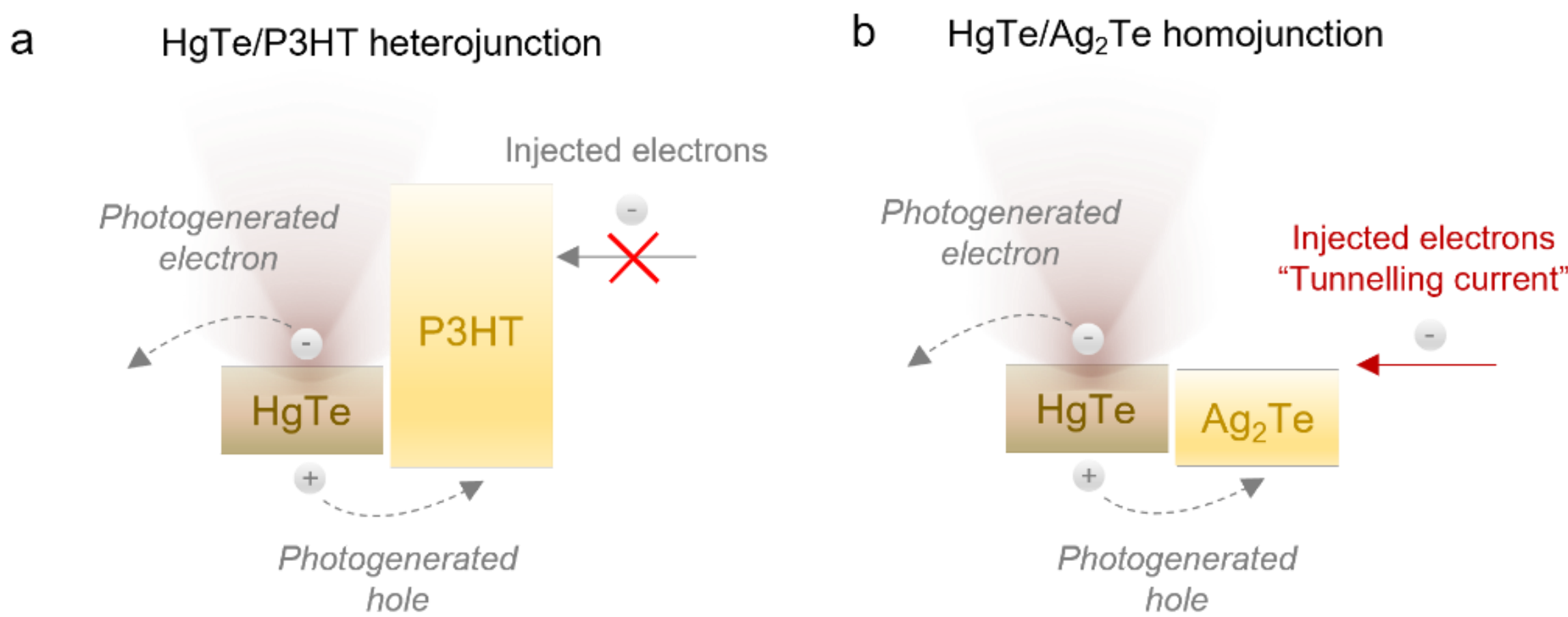

Fig. S5. Comparison of Heterojunction and Homojunction.

Energy band diagram of (**a**) HgTe CQDs/poly(3-hexylthiophene-2,5-diyl) (P3HT) heterojunction and (**b**) HgTe CQDs/silver telluride (Ag_2Te) homojunction.

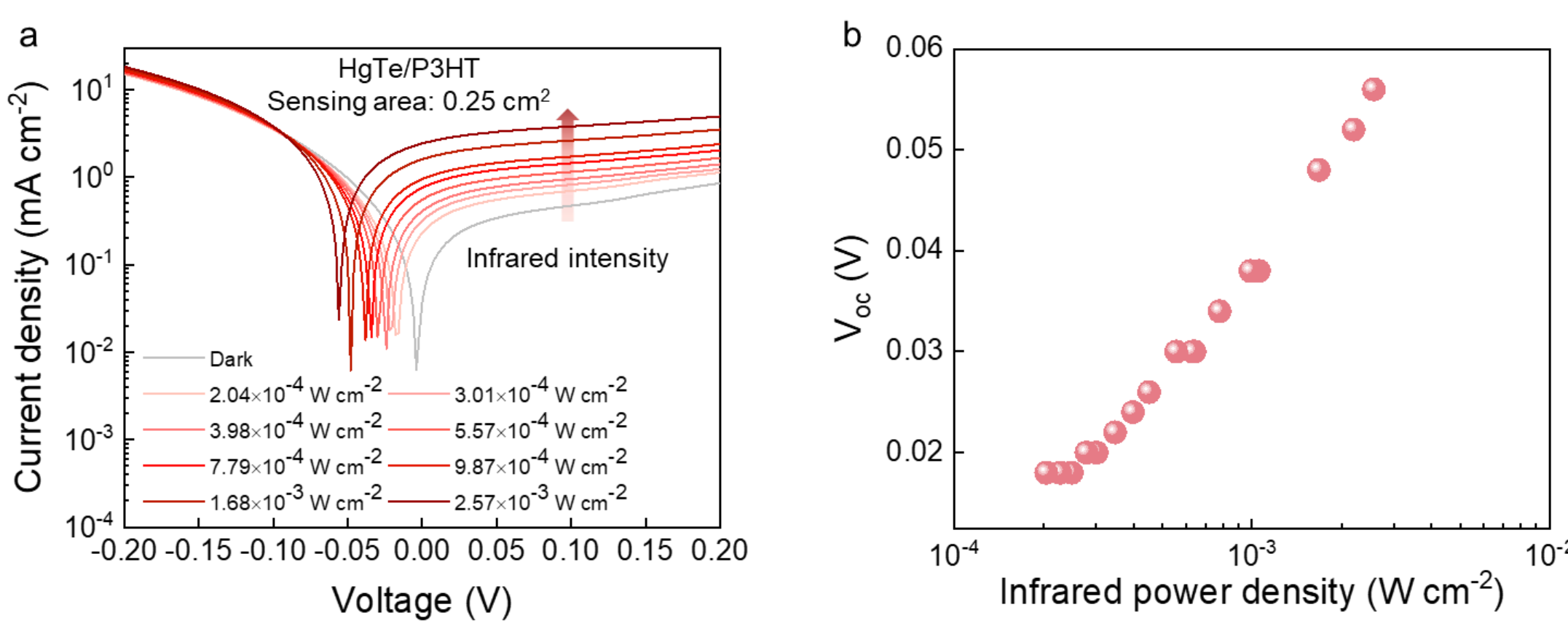

Fig. S6. Infrared Photodetection.

(**a**) Current density versus voltage curves of HgTe/P3HT photodetector (PD) without and with infrared of different power densities. (**b**) Open-circuit voltage (V_{OC}) versus infrared power density curves of the HgTe/P3HT PD.

Responsivity ($\Re$, units of A W^{-1}) of photodetectors:

$$\Re = \frac{I_{ph}}{P} \tag{S1}$$

where I_{ph} is the photocurrent and P is the input optical power.

External quantum efficiency (EQE, units of %) of photodetectors:

$$EQE = \frac{1240}{\lambda}\Re \tag{S2}$$

where λ is the wavelength of the infrared, and $\Re$ is the responsivity.

Detectivity (D^*, units of $cm \cdot Hz^{1/2} \cdot W^{-1}$ or Jones) of photodetectors:

$$D^* = \frac{\sqrt{A}}{I_n} \Re \tag{S3}$$

where A is the sensing area, I_n is the spectral noise density, and $\Re$ is the responsivity.

Section S3. Multicolor Emission

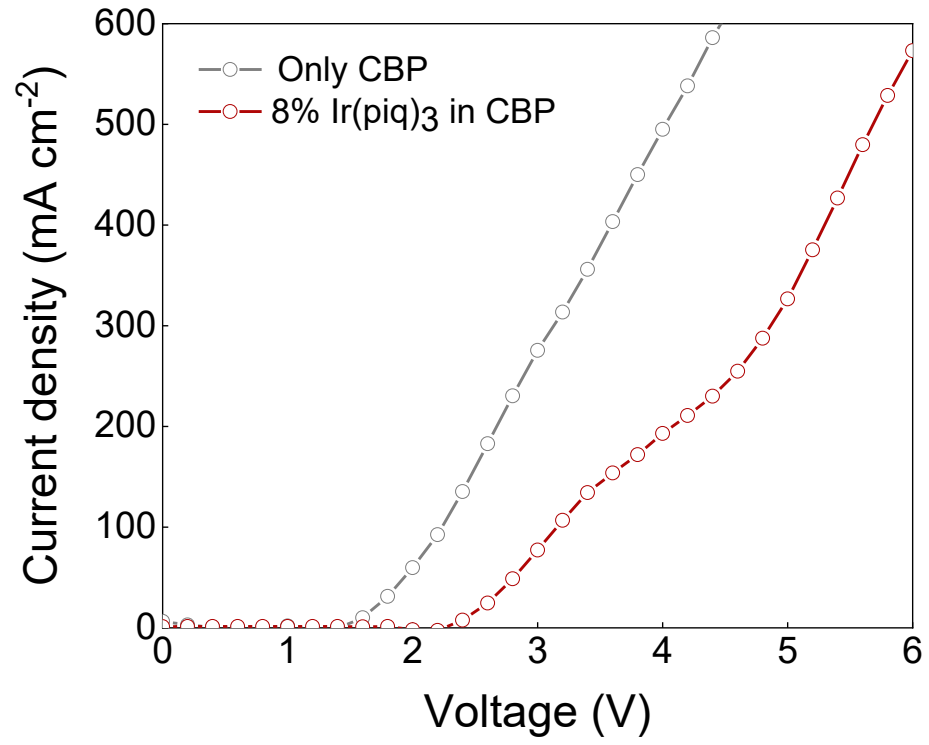

Fig. S7. Hole-only Devices.
The current density-voltage characteristic of hole-only devices.

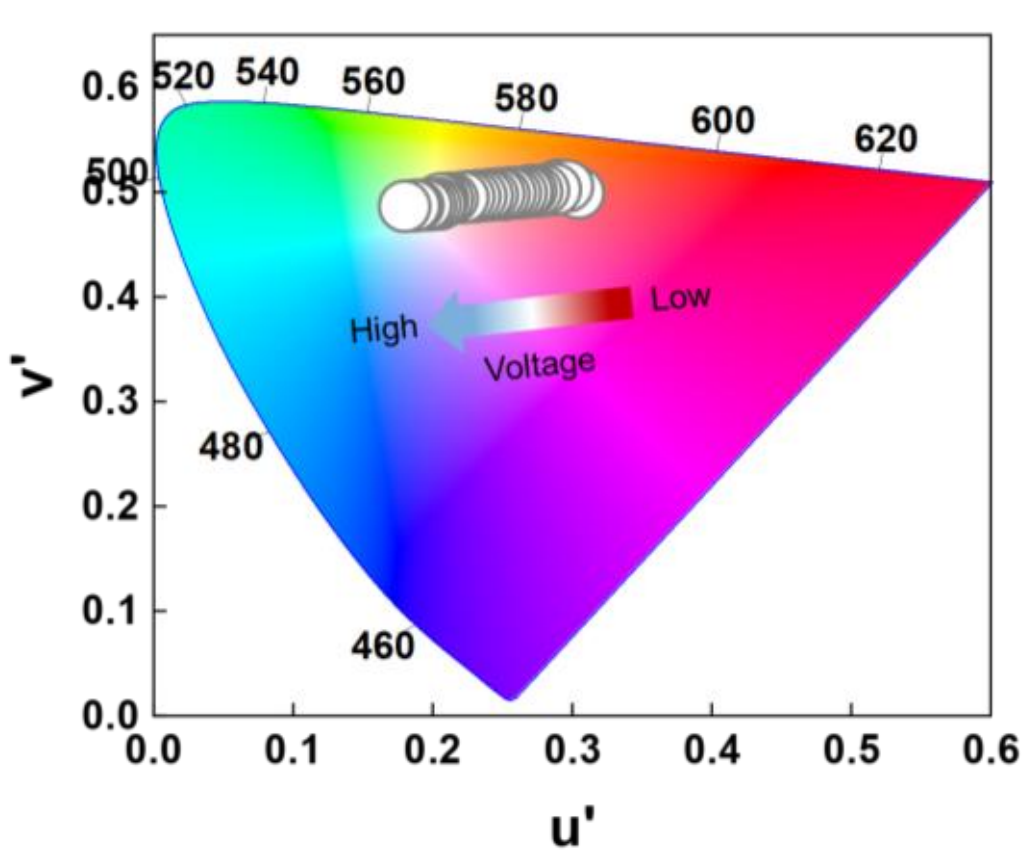

Fig. S8. Multicolor Emission.
Evolution of the *CIE 1976* chromaticity coordinates of the multicolor OLED with increasing voltage.

The influence of the host materials of the red and cyan-emitting layer (EML) has been explored (**Fig. S9** and **S10**). When the host material of the red EML is TCTA, the red light component emitted by the device is almost non-existent; when the host material of the red EML is CBP, the red light emission is better. When the host material of the cyan EML is selected as CzPhPy, the cyan light component emitted by the device is very small, and thus the device cannot achieve the color change from red to cyan.

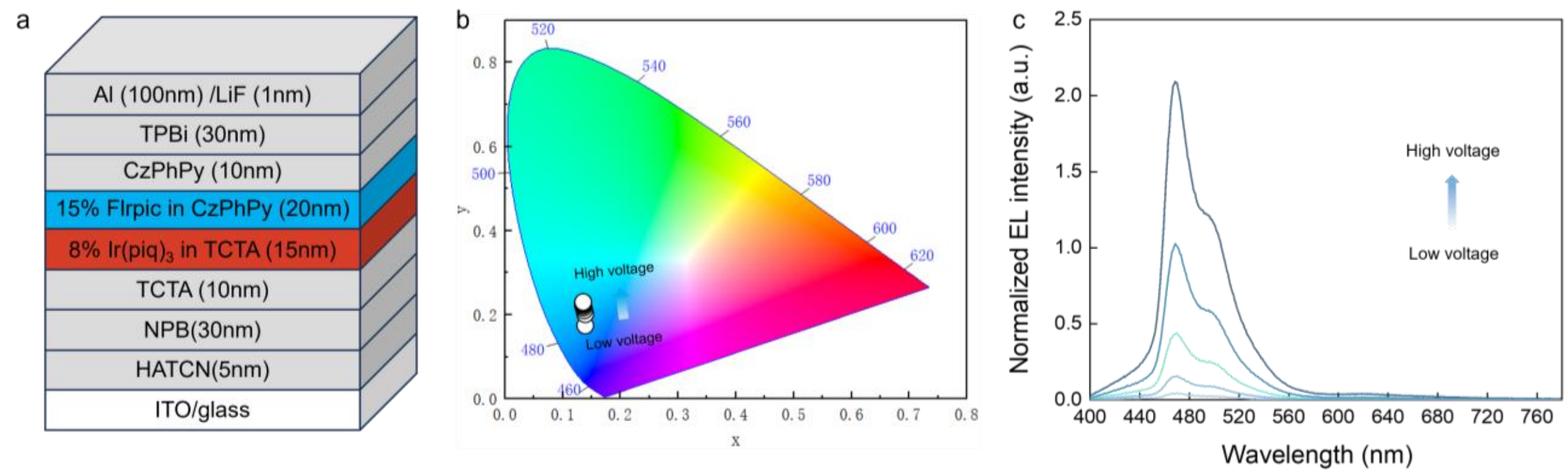

Fig. S9. Control Experiment.
(**a**) Device structure diagram of the control OLED of the 8% Ir(piq)$_3$ in TCTA (15 nm), 15% Flrpic in CzPhPy (20 nm). Evolution of (**b**) *CIE 1931* chromaticity coordinates and (**c**) normalized electroluminescence (*EL*) spectra of the control OLED of the 8% Ir(piq)$_3$ in TCTA (15 nm), 15% Flrpic in CzPhPy (20 nm) with increasing voltage.

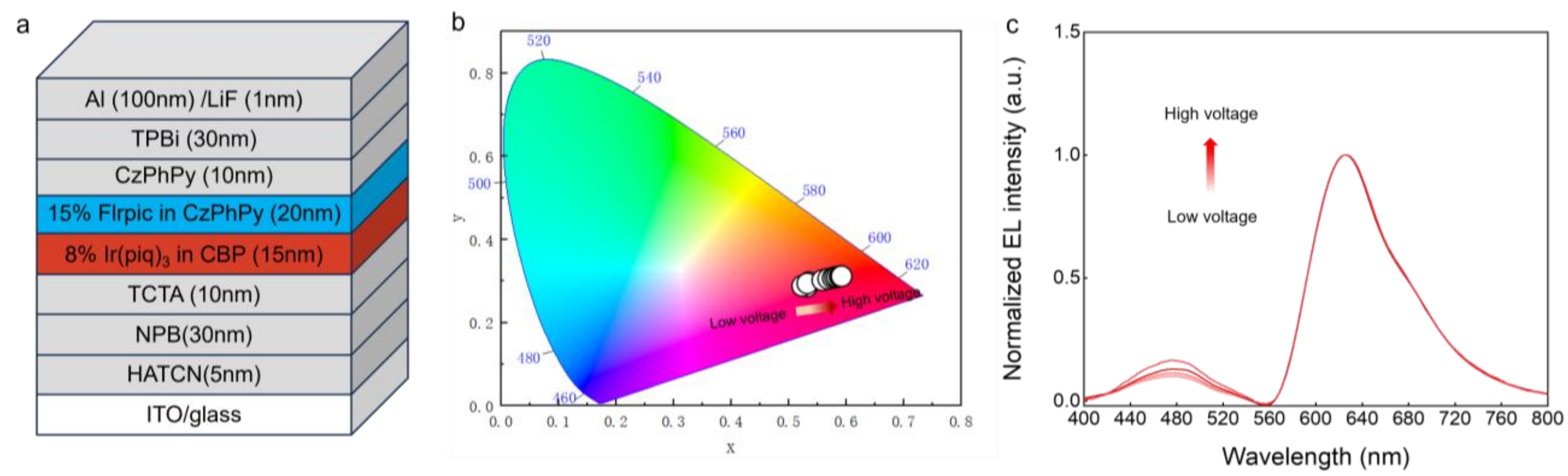

Fig. S10. Control Experiment.
(**a**) Device structure diagram of the control OLED of the 8% Ir(piq)$_3$ in CBP (15 nm), 15% Flrpic in CzPhPy (20 nm). Evolution of (**b**) *CIE 1931* chromaticity coordinates and (**c**) normalized *EL* spectra of the control OLED of the 8% Ir(piq)$_3$ in CBP (15 nm), 15% Flrpic in CzPhPy (20 nm) with increasing voltage.

The thicknesses of the red and cyan EML have been explored, and their effects on exciton allocation with control groups of 5 nm red EML/20 nm cyan EML and 15 nm red EML/10 nm cyan EML have been studied. When the thickness of the red EML decreases from 15 nm to only 5 nm, the holes cross the energy barrier to the cyan EML more easily. Thus, the *EL* peak of the cyan component is larger than that of the red component during the whole voltage process, leading to the main cyan emission (**Fig. S11**). In addition, the primary emission is red, with a red *EL* peak much larger than the cyan peak, as the thickness of the cyan EML decreases from 20 nm to 10 nm (**Fig. S12**). Owing to the unbalanced red and cyan emission, the range of color variation with the voltage of the control groups is much smaller than the optimal thickness of the 15 nm red EML/20 nm cyan EML.

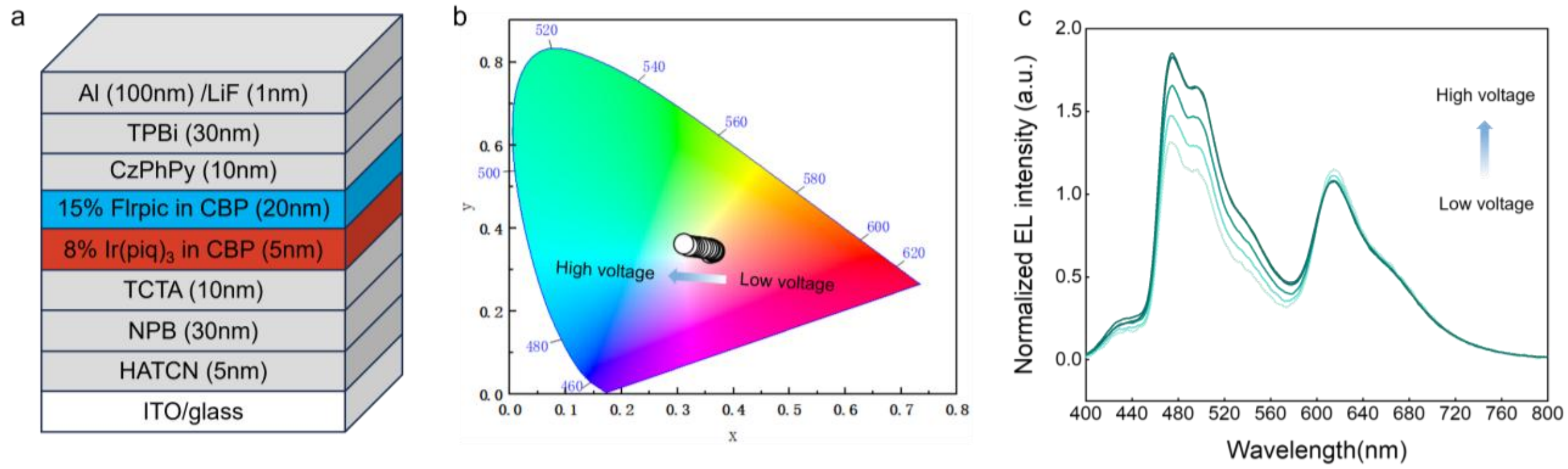

Fig. S11. Control Experiment.

(**a**) Device structure diagram of the control OLED of the 5 nm red EML/ 20 nm cyan EML. Evolution of (**b**) *CIE 1931* chromaticity coordinates and (**c**) normalized *EL* spectra of the control OLED of 5 nm red EML/ 20 nm cyan EML with increasing voltage.

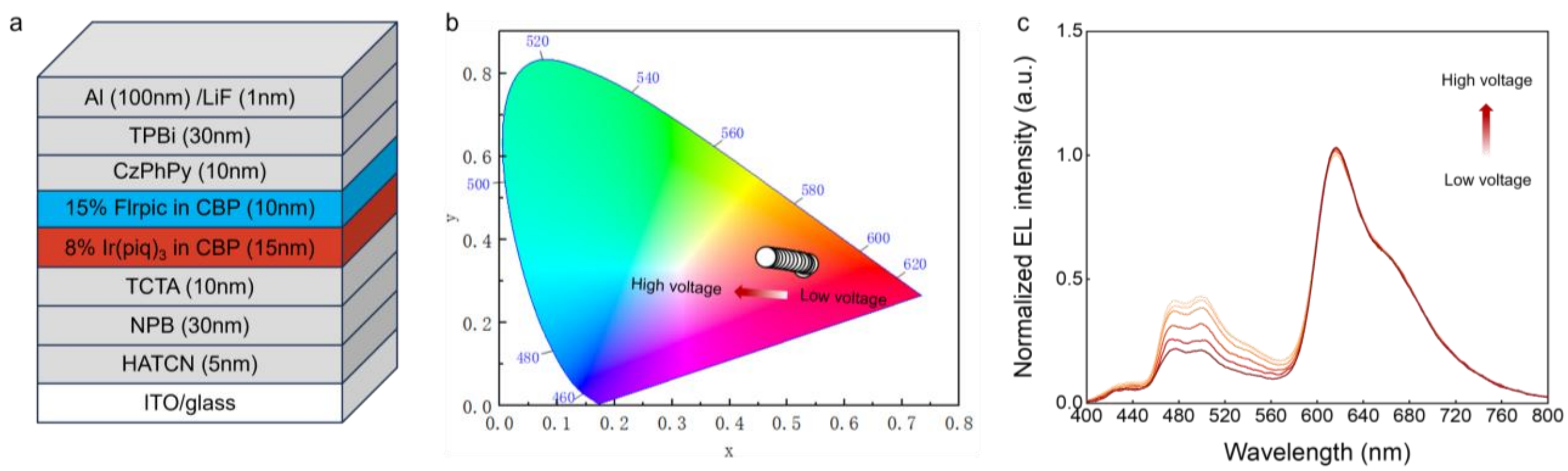

Fig. S12. Control Experiment.

(**a**) Device structure diagram of the control OLED of 15 nm red EML/ 10 nm cyan EML. Evolution of (**b**) *CIE 1931* chromaticity coordinates and (**c**) normalized *EL* spectra of the control OLED of 15 nm red EML/ 10 nm cyan EML with increasing voltage.

External quantum efficiency (*EQE*, units of %) of OLED:

$$\eta_{EQE} = \frac{\pi L e}{K_m h c J} \frac{\int I(\lambda)\lambda d\lambda}{\int I(\lambda)V(\lambda)d\lambda} \tag{S4}$$

where e is the electron charge, h is the Planck constant, c is the velocity of light, K_m = 683 lm W^{-1} is the maximum luminous efficacy, J is the current density, L is the luminance, λ is the wavelength, $I(\lambda)$ is the relative *EL* intensity at a wavelength λ, $V(\lambda)$ is the normalized photonic spectral response function.

Section S4. Infrared-to-Full-Color Visible Upconversion

Infrared photon-to-visible photon upconversion efficiency ($\eta_{p/p}$, units of %):

$$\eta_{p/p} = \frac{N_e}{N_i} = \frac{P_e / \frac{hc}{\lambda_e}}{P_i / \frac{hc}{\lambda_i}} \tag{S5}$$

where N_e and N_i are the number of emitted visible photons and incident infrared photons, respectively, h is the Planck constant, c is the speed of light, λ_e and λ_i are the wavelengths of the emitted visible light and incident infrared light, respectively, P_e and P_i are the power of the emitted visible photons and the incident infrared photons, respectively.

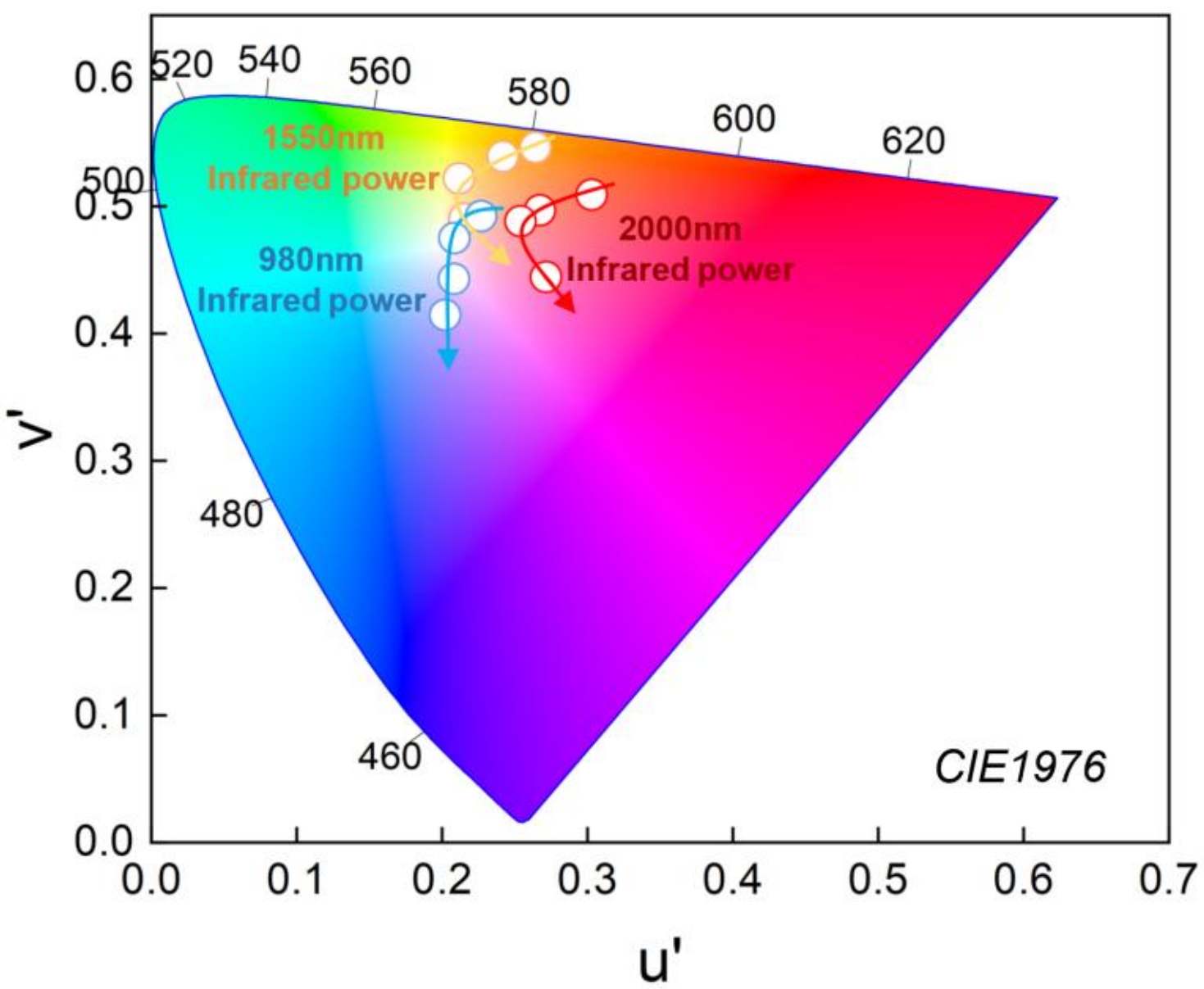

Fig. S13. Infrared-to-Full-Color Visible Upconversion.

Evolution of *CIE 1976* chromaticity coordinates of infrared-to-full-color upconverter with increasing infrared power densities for different infrared wavelengths of 980, 1550, and 2000 nm.

Color difference of two colors in *CIE* $L^*a^*b^*$ color space (L_1^*, a_1^*, b_1^*) and (L_2^*, a_2^*, b_2^*)(*50*):

$$\Delta E_{ab}^* = \sqrt{(\Delta L^*)^2 + (\Delta a^*)^2 + (\Delta b^*)^2} = \sqrt{(L_2^* - L_1^*)^2 + (a_2^* - a_1^*)^2 + (b_2^* - b_1^*)^2} \tag{S6}$$

Section S5. Infrared Visualization Wearable Eyeglass

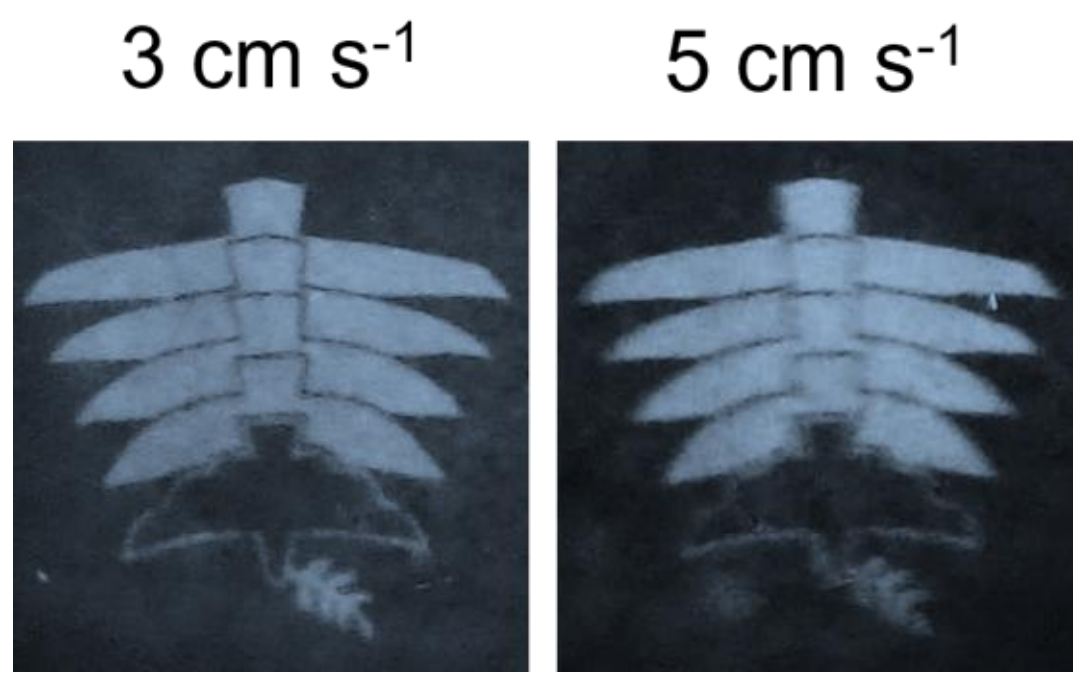

Fig. S14. Infrared Visualization Wearable Eyeglass.
Dynamic infrared upconversion imaging when the object is moving with various motion velocities of 3 cm s^{-1} and 5 cm s^{-1}.

The infrared signal propagating through the 0.4 mm spacing between the points of the mask is precisely upconverted. The image resolution is estimated to be 64 dpi. This resolution is primarily limited by the size and spacing of the mask used in our current laboratory setup. By employing a mask with finer features and smaller spacing, the upconverter has the potential to achieve higher imaging resolution.

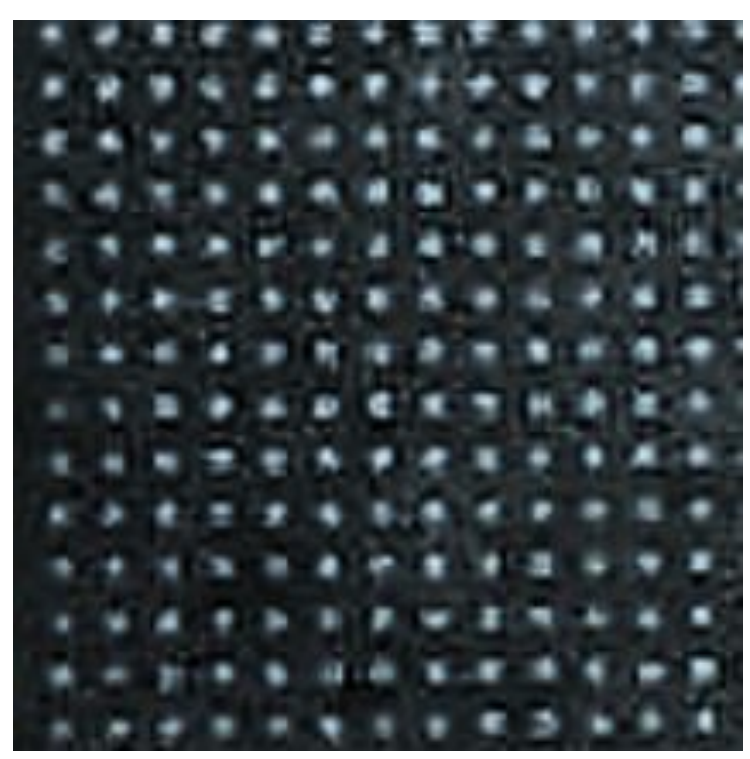

Fig. S15. Infrared Visualization Wearable Eyeglass.
The infrared upconversion imaging is performed through a dot matrix array-shaped mask with 0.4 mm spacing between the points.

Optimization strategies to decrease the operation voltages of the upconverter.
While the current prototype demonstrates the feasibility of multispectral infrared visualization in a wearable form, reducing the operational voltage remains a critical objective for enhancing portability and long-term stability. To achieve this, several optimization strategies are planned for the next generation of the device:
First, for the infrared detecting unit, designing a band-engineered heterojunction with optimized energy alignment at the hole-blocking/transport layers will enhance the built-in potential and minimize non-radiative recombination, thereby increasing the open-circuit voltage. Additionally,

serial stacking of multiple photovoltaic units could cumulatively increase the total open-circuit voltage to effectively reach the turn-on threshold of the light-emitting unit.

Second, the visible emitting unit will be optimized by utilizing low-dimensional materials, such as perovskite or quantum dots, which are known for their ultra-low turn-on voltages. Implementing highly efficient co-host systems will also ensure balanced charge transport and high emission efficiency at lower driving biases.

Third, interface engineering between the detector and emitter is paramount. By introducing specialized interlayers, we can eliminate the potential barriers that currently hinder efficient carrier injection at the interface from the detection unit to the emitting unit.

Section S6. Infrared Perception Retinal Photoreceptor

Behaviors of light-sensitive proteins bound to upconverters when excitation at 1550 nm or 2000 nm

Channelrhodopsin-2 (ChR2) was selected to verify the potential of upconverters for neural-level infrared vision due to its status as the most widely adopted light-sensitive protein in optogenetics (*46, 47*). ChR2 is a blue-light-sensitive opsin with a peak sensitivity around 470 nm and a relatively low activation light power threshold (0.1 mW mm^{-2}). Our upconverter under 980 nm excitation generates cyan/blue light with a peak at ~470 nm, which perfectly aligns with the ChR2 activation spectrum.

However, when excitation of 1550 and 2000 nm, the upconverter emits yellow or red light. ChR2 cannot be activated by these colors. While red- or yellow-sensitive opsins often require higher excitation power densities (e.g., ReaChR with 590 nm of 0.5~2 mW mm^{-2} activation) or exhibit slower kinetics that could complicate the physiological response (*46, 47*), which are not chosen in this work. We envision that if other suitable opsins sensitive to the specific visible emission bands (e.g., yellow or red) produced under 1550 nm and 2000 nm excitation are employed, we would expect to observe neural activation and behaviors similar to the 980 nm-ChR2 system. The current implementation using ChR2 serves as a robust proof-of-concept, demonstrating that our upconversion architecture can successfully translate infrared spectral information into biological signals.

REFERENCES

1. M. T. H. Do, S. H. Kang, T. Xue, H. Zhong, H. W. Liao, D. E. Bergles, K. W. Yau, Photon capture and signalling by melanopsin retinal ganglion cells. *Nature* **457**, 281–287 (2009).

2. D.-G. Luo, W. W. S. Yue, P. Ala-Laurila, K.-W. Yau, Activation of visual pigments by light and heat. *Science* **332**, 1307–1312 (2011).

3. D. Nelidova, R. K. Morikawa, C. S. Cowan, Z. Raics, D. Goldblum, H. P. N. Scholl, T. Szikra, A. Szabo, D. Hillier, B. Roska, Restoring light sensitivity using tunable near-infrared sensors. *Science* **368**, 1108–1113 (2020).

4. G. Mu, C. Bi, J. Zou, Y. Liu, Q. Hao, X. Tang, Hyperspectral quantum-dot image sensors via in-pixel reconfigurable band-alignment. *Nat. Photonics* **20**, 523–531 (2026).

5. H. Ma, K. Huang, J. Fang, Z. He, Y. Liang, H. Zeng, Mid-infrared single-pixel imaging via two-photon optical encoding. *PhotoniX* **6**, 34 (2025).

6. S. Chen, A. Z. Weitemier, X. Zeng, L. He, X. Wang, Y. Tao, A. J. Y. Huang, Y. Hashimotodani, M. Kano, H. Iwasaki, L. K. Parajuli, S. Okabe, D. B. L. Teh, A. H. All, I. Tsutsui-Kimura, K. F. Tanaka, X. Liu, T. J. McHugh, NIR deep brain stimulation via upconversion nanoparticle-mediated optogenetics. *Science* **359**, 679–684 (2018).

7. G. Bao, D. Jin, Nanoparticles give mice infrared vision. *Nat. Photonics* **13**, 304–305 (2019).

8. Y. Ma, J. Bao, Y. Zhang, Z. Li, X. Zhou, C. Wan, L. Huang, Y. Zhao, G. Han, T. Xue, Mammalian near-infrared image vision through injectable and self-powered retinal nanoantennae. *Cell* **177**, 243–255.e15 (2019).

9. Y. Ma, Y. Chen, S. Wang, Z. H. Chen, Y. Zhang, L. Huang, X. Zhang, F. Yin, Y. Wang, M. Yang, Z. Li, K. Huang, X. Fang, Z. Li, M. Wang, W. Liu, J. N. Li, L. Li, H. Zhao, M. Wei, Y. Shi, R. Liu, M. Zhang, J. Chen, J. Shen, J. Meng, Y. Yang, F. Zhang, X. Gong, G. Han, T. Xue, Near-infrared spatiotemporal color vision in humans enabled by upconversion contact lenses. *Cell* **188**, 3375–3388.e18 (2025).

10. S. Wang, K. Liang, H. Zhao, M. Wu, J. He, P. Wei, Z. Ding, J. Li, X. Huang, Y. Ren, Electronic structure formed by Y_2O_3-doping in lithium position assists improvement of charging-voltage for high-nickel cathodes. *Nat. Commun.* **16**, 1 (2025).

11. S. Wang, C. Jiang, Y. Yu, Z. Zhang, R. Quhe, R. Yang, Y. Tian, X. Chen, W. Fan, Y. Niu, B. Yan, C. Jiang, Y. Wang, Z. Wang, C. Liu, W. Hu, J. Zhang, P. Zhou, Tellurium nanowire retinal nanoprosthesis improves vision in models of blindness. *Science* **388**, eadu2987 (2025).

12. H. Ding, L. Lu, Z. Shi, D. Wang, L. Li, X. Li, Y. Ren, C. Liu, D. Cheng, H. Kim, N. C. Giebink, X. Wang, L. Yin, L. Zhao, M. Luo, X. Sheng, Microscale optoelectronic infrared-to-visible upconversion devices and their use as injectable light sources. *Proc. Natl. Acad. Sci. U.S.A.* **115**, 6632–6637 (2018).

13. C.-J. Shih, Y.-C. Huang, T.-Y. Wang, C.-W. Yu, I.-S. Hsu, A. K. Akbar, J.-Y. Lin, S. Biring, J.-H. Lee, S.-W. Liu, Transparent organic upconversion devices displaying high-resolution, single-pixel, low-power infrared images perceived by human vision. *Sci. Adv.* **9**, eadd7526 (2023).

14. Y. Chen, Y. Zheng, J. Wang, X. Zhao, G. Liu, Y. Lin, Y. Yang, L. Wang, Z. Tang, Y. Wang, Y. Fang, W. Zhang, X. Zhu, Ultranarrow-bandgap small-molecule acceptor enables sensitive SWIR detection and dynamic upconversion imaging. *Sci. Adv.* **10**, eadm9631 (2024).

15. J. Chen, J. Tao, D. Ban, M. G. Helander, Z. Wang, J. Qiu, Z. Lu, Hybrid organic/inorganic optical up-converter for pixel-less near-infrared imaging. *Adv. Mater.* **24**, 3138–3142 (2012).

16. W. Zhou, Y. Shang, F. P. García de Arquer, K. Xu, R. Wang, S. Luo, X. Xiao, X. Zhou, R. Huang, E. H. Sargent, Z. Ning, Solution-processed upconversion photodetectors based on quantum dots. *Nat. Electron.* **3**, 251–258 (2020).

17. H. Yu, D. Kim, J. Lee, S. Baek, J. Lee, R. Singh, F. So, High-gain infrared-to-visible upconversion light-emitting phototransistors. *Nat. Photonics* **10**, 129–134 (2016).

18. K. J. Russella, I. Appelbaum, H. Temkin, C. H. Perry, V. Narayanamurti, M. P. Hanson, A. C. Gossard, Room-temperature electro-optic up-conversion via internal photoemission. *Appl. Phys. Lett.* **82**, 2960–2962 (2003).

19. H. Beneking, N. Grote, M. N. Svilans, Monolithic GaAlAs/GaAs infrared-to-visible wavelength converter with optical power amplification. *IEEE Trans. Electron Devices* **28**, 404–407 (1981).

20. C.-J. Shih, Y.-Z. Li, M.-Z. Li, S. Biring, B.-C. Huang, C.-W. Liu, T.-H. Yeh, D. Luo, J.-H. Lee, Y.-H. Huang, K.-T. Wong, S.-W. Liu, Transparent organic upconversion device targeting high- grade infrared visual image. *Nano Energy* **86**, 106043 (2021).

21. K. Strassel, A. Kaiser, S. Jenatsch, A. C. Véron, S. B. Anantharaman, E. Hack, M. Diethelm, F. Nüesch, R. Aderne, C. Legnani, S. Yakunin, M. Cremona, R. Hany, Squaraine dye for a visibly transparent all-organic optical upconversion device with sensitivity at 1000 nm. *ACS Appl. Mater. Interfaces* **10**, 11063–11069 (2018).

22. D. Yang, X. Zhou, D. Ma, A. Vadim, T. Ahamad, S. M. Alshehri, Near infrared to visible light organic up-conversion devices with photon-to-photon conversion efficiency approaching 30%. *Mater. Horiz.* **5**, 874–882 (2018).

23. X. Hu, G. Xiao, Y. Li, S. E. Wu, Q. Chen, N. Li, X. Sui, Infrared-light visualization by organic upconversion devices. *ACS Appl. Electron. Mater.* **5**, 5378–5385 (2023).

24. N. Li, N. Eedugurala, D. S. Leem, J. D. Azoulay, T. N. Ng, Organic upconversion imager with dual electronic and optical readouts for shortwave infrared light detection. *Adv. Funct. Mater.* **31**, 2100565 (2021).

25. X. Du, J. Han, Z. He, C. Han, X. Wang, J. Wang, Y. Jiang, S. Tao, Efficient organic upconversion devices for low energy consumption and high-quality noninvasive imaging. *Adv. Mater.* **33**, e2102812 (2021).

26. G. Mu, Y. Lin, K. Fu, X. Tang, Infrared visualized snakes-inspired artificial vision systems with CMOS sensors-integrated upconverters. *Light Sci. Appl.* **14**, 282 (2025).

27. N. Zhang, H. Tang, K. Shi, W. Wang, W. Deng, B. Xu, K. Wang, X. W. Sun, High-performance all-solution-processed quantum dot near-infrared-to-visible upconversion devices for harvesting photogenerated electrons. *Appl. Phys. Lett.* **115**, 221103 (2019).

28. Q. Xu, X. Yang, J. J. Liu, F. Li, R. Chang, L. Wang, A. Q. Wang, Z. Wu, H. Shen, Z. Du, Elaborating the interplay between the detecting unit and emitting unit in infrared quantum dot up-conversion photodetectors. *Nanoscale* **15**, 8197–8203 (2023).

29. H. Tang, K. Shi, N. Zhang, Z. Wen, X. Xiao, B. Xu, H. Butt, Z. Pikramenou, K. Wang, X. W. Sun, Up-conversion device based on quantum dots with high-conversion efficiency over 6%. *IEEE Access* **8**, 71041–71049 (2020).

30. G. Mu, T. Rao, S. Zhang, C. Wen, M. Chen, Q. Hao, X. Tang, Ultrasensitive colloidal quantum-dot upconverters for extended short-wave infrared. *ACS Appl. Mater. Interfaces* **14**, 45553–45561 (2022).

31. G. Mu, T. Rao, Y. Qi, S. Ma, Q. Hao, M. Chen, X. Tang, Color-tunable organic light-emitting displays for interactive multi-signal visualization. *Adv. Funct. Mater.* **33**, 2301280 (2023).

32. B. Roska, J. A. Sahel, Restoring vision. *Nature* **557**, 359–367 (2018).

33. R. A. Pearson, A. C. Barber, M. Rizzi, C. Hippert, T. Xue, E. L. West, Y. Duran, A. J. Smith, J. Z. Chuang, S. A. Azam, U. F. O. Luhmann, A. Benucci, C. H. Sung, J. W. Bainbridge, M. Carandini, K. W. Yau, J. C. Sowden, R. R. Ali, Restoration of vision after transplantation of photoreceptors. *Nature* **485**, 99–103 (2012).

34. E. Lhuillier, S. Keuleyan, P. Guyot-Sionnest, Optical properties of HgTe colloidal quantum dots. *Nanotechnology* **23**, 175705 (2012).

35. H. Liu, S. Keuleyan, P. Guyot-Sionnest, N- and p-type HgTe quantum dot films. *J. Phys. Chem. C* **116**, 1344–1349 (2012).

36. A. Al-otaify, S. V. Kershaw, S. Gupta, A. L. Rogach, G. Allan, C. Delerue, D. J. Binks, Multiple exciton generation and ultrafast exciton dynamics in HgTe colloidal quantum dots. *Phys. Chem. Chem. Phys.* **15**, 16864–16873 (2013).

37. M. C. Beard, Multiple exciton generation in semiconductor quantum dots. *J. Phys. Chem. Lett.* **2**, 1282–1288 (2011).

38. G. Mu, X. Zheng, Y. Tan, Y. Liu, Q. Hao, K. Weng, X. Tang, Colloidal quantum-dot heterojunction imagers for room-temperature thermal imaging. *Adv. Mater.* **37**, 2416877 (2025).

39. M. M. Ackerman, X. Tang, P. Guyot-Sionnest, Fast and sensitive colloidal quantum dot mid-wave infrared photodetectors. *ACS Nano* **12**, 7264–7271 (2018).

40. X. Tang, M. M. Ackerman, M. Chen, P. Guyot-Sionnest, Dual-band infrared imaging using stacked colloidal quantum dot photodiodes. *Nat. Photonics* **13**, 277–282 (2019).

41. T. Qin, G. Mu, P. Zhao, Y. Tan, Y. Liu, S. Zhang, Y. Luo, Q. Hao, M. Chen, X. Tang, Mercury telluride colloidal quantum-dot focal plane array with planar p-n junctions enabled by in situ electric field-activated doping. *Sci. Adv.* **9**, eadg7827 (2023).

42. C. Zhang, D. Zhang, Z. Bin, Z. Liu, Y. Zhang, H. Lee, J. H. Kwon, L. Duan, Color-tunable all-fluorescent white organic light-emitting diodes with a high external quantum efficiency over 30% and extended device lifetime. *Adv. Mater.* **34**, 2103102 (2022).

43. T. H. Kwon, H. Bin Kim, D. G. Kwak, D. Hahm, S. Yoo, B. S. Kim, W. K. Bae, M. S. Kang, Quantum dot-based three-stack tandem near-infrared-to-visible optoelectric upconversion devices. *ACS Nano* **18**, 21957–21965 (2024).

44. S. Hecht, The visual discrimination of intensity and the Weber-Fechner law. *J. Gen. Physiol.* **7**, 235–267 (1924).

45. Y. Liu, G. Wang, F. Feng, M. Zhanghu, Z. Yuan, Z. Li, K. Xu, H. S. Kwok, Z. Liu, Ultra-low-defect homoepitaxial micro-LEDs with enhanced efficiency and monochromaticity for high-PPI AR/MR displays. *PhotoniX* **5**, 23 (2024).

46. X. Lin, Y. Wang, X. Chen, R. Yang, Z. Wang, J. Feng, H. Wang, K. W. C. Lai, J. He, F. Wang, P. Shi, Multiplexed optogenetic stimulation of neurons with spectrum-selective upconversion nanoparticles. *Adv. Healthc. Mater.* **6**, 10.1002/adhm.201700446 (2017).

47. Y. Wang, X. Lin, X. Chen, X. Chen, Z. Xu, W. Zhang, Q. Liao, X. Duan, X. Wang, M. Liu, F. Wang, J. He, P. Shi, Tetherless near-infrared control of brain activity in behaving animals using fully implantable upconversion microdevices. *Biomaterials* **142**, 136–148 (2017).

48. Y. Wang, K. Xie, H. Yue, X. Chen, X. Luo, Q. Liao, M. Liu, F. Wang, P. Shi, Flexible and fully implantable upconversion device for wireless optogenetic stimulation of the spinal cord in behaving animals. *Nanoscale* **12**, 2406–2414 (2020).

49. G. Mu, Y. Tan, C. Bi, Y. Liu, Q. Hao, X. Tang, Visible to mid-wave infrared PbS/HgTe colloidal quantum dot imagers. *Nat. Photonics* **18**, 1147–1154 (2024).

50. B. Hill, T. Roger, F. W. Vorragen, Comparative analysis of the quantization of color spaces on the basis of the CIELAB color-difference formula. *ACM Trans. Graph.* **16**, 109–154 (1997).
